# Sulfonate derivatives bearing an amide unit: design, synthesis and biological activity studies

**DOI:** 10.1186/s13065-024-01151-0

**Published:** 2024-03-06

**Authors:** You-hua Liu, Chang-kun Li, Mao-yu Nie, Fa-li Wang, Xiao-li Ren, Lin-hong Jin, Xia Zhou

**Affiliations:** National Key Laboratory of Green Pesticide, Key Laboratory of Green Pesticide and Agricultural Bioengineering, Ministry of Education, Center for R&D of Fine Chemicals of Guizhou University, Guiyang, 550025 China

**Keywords:** Sulfonate derivatives, Synthesis, Pesticides, Biological activity

## Abstract

Pest disasters which occurs on crops is a serious problem that not only cause crop yield loss or even crop failure but can also spread a number of plant diseases.Sulfonate derivatives have been widely used in insecticide and fungicide research in recent years. On this basis, a series of sulfonate derivatives bearing an amide unit are synthesized and the biological activities are evaluated. The bioassay results showed that compounds **A**_**8**_, **A**_**13**_, **A**_**16**_, **B**_**1**_, **B**_**3**_, **B**_**4**_, **B**_**5**_, **B**_**10**_, **B**_**12 − 20**_, **C**_**3**_, **C**_**5**_, **C**_**9**_, **C**_**10**_, **C**_**14**_, **C**_**15**_, **C**_**17**_ and **C**_**19**_ showed 100% activity at a concentration of 500 *µ*g/mL against the *Plutella xylostella* (*P. xylostella*). Among them, **B**_**15**_ which contains a thiadiazole sulfonate structure still shows 100% activity at 50 *µ*g/mL concentration against *P. xylostella* and had the lowest median lethal concentration (LC_50_) (7.61 *µ*g/mL) among the target compounds. Further mechanism studies are conducted on compounds with better insecticidal activity. Molecular docking results shows that **B**_**15**_ formed hydrophobic interactions π-π and hydrogen bonds with the indole ring of Trp532 and the carboxyl group of Asp384, respectively, with similar interaction distances or bond lengths as those of diflubenzuron. Moreover, chitinase inhibition assays are performed to further demonstrate its mode of action. In addition, the anti-bacterial activity of the series of compounds is also tested and the results showed that the series of compounds has moderate biological activity against *Xanthomonas oryzae* pv. *oryzae* (*Xoo*) and *Xanthomonas oryzae* pv. *oryzicola* (*Xoc*), with inhibition rates of 91%, 92% and 92%, 88% at the concentration of 100 *µ*g/mL, respectively. Our study indicates that **B**_**15**_ can be used as a novel insecticide for crop protection.

**Graphical Abstract:**

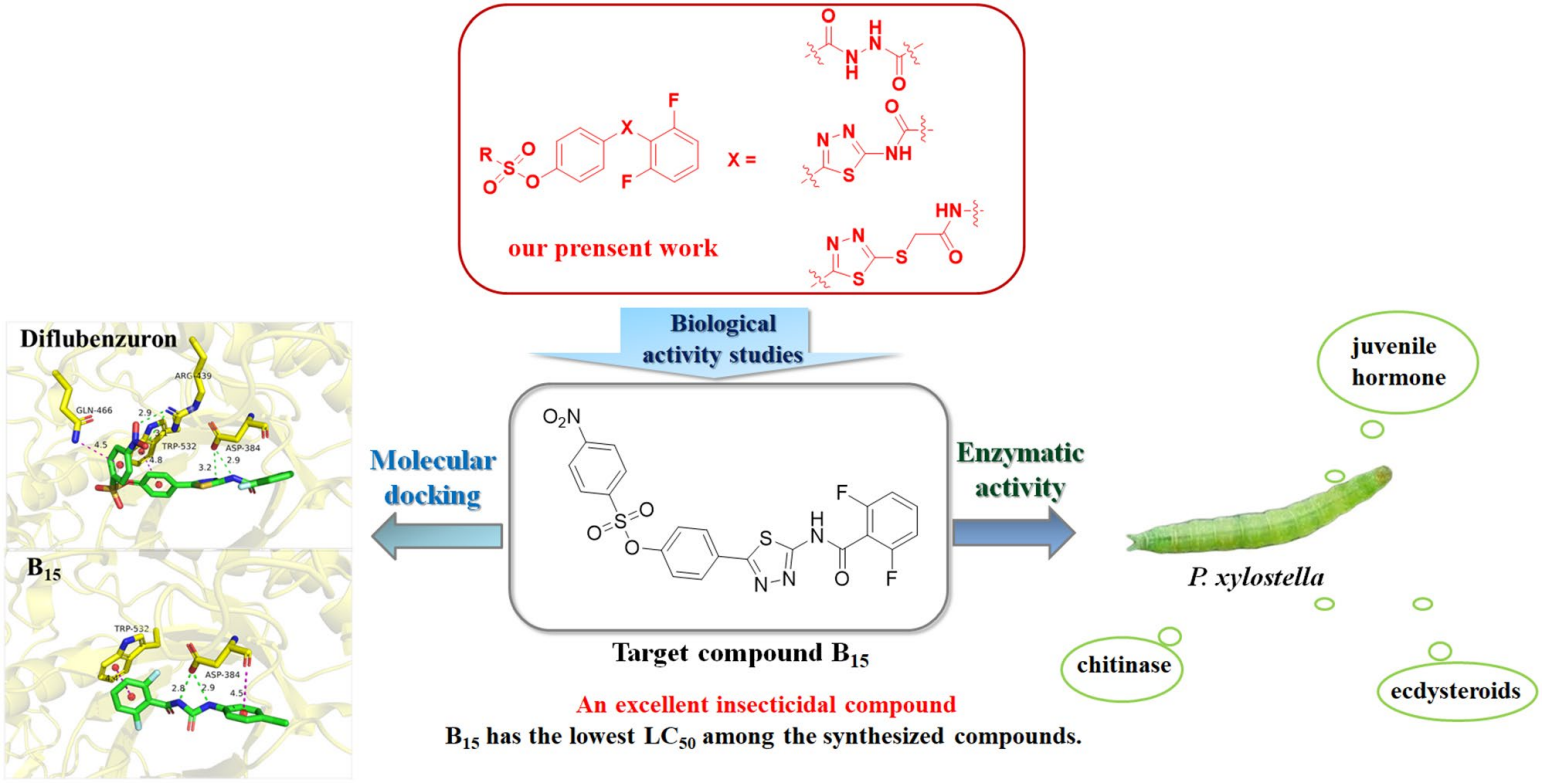

**Supplementary Information:**

The online version contains supplementary material available at 10.1186/s13065-024-01151-0.

## Introduction

Agricultural pests are the most important impact factors that threaten crops. They often cause serious quality and yield losses in agricultural production [1, 2]. Become potential “carriers” while feeding on susceptible plants, spreading more serious diseases to the plants. Among agricultural pests, the *P. xylostella* is one of the most challenging pests to control, infesting cruciferous crops and causing up to $4.5 billion in losses annually [3]. Since crops are threatened not only by agricultural pests but also by crop pathogens, the focus of addressing these issues is on effective disease and pest management. Therefore, different types of pesticides such as fungicides/bactericides, insecticides, are applied to manage the corresponding pests and diseases [4]. However, 4.6 million tons of pesticides are used worldwide every year, with about 90% of them unable to be used properly and ending up in vulnerable farming systems, leading to an excessive build-up of harmful residues in crops [5–11]. Hence, the development of novel insecticidal agents is particularly important to reduce impacts on agroecosystem diversity.

**Fig. 1:**
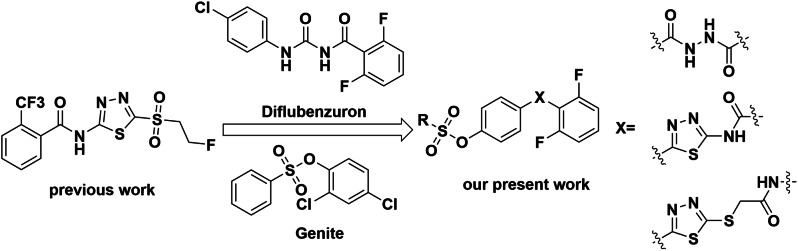
Design strategy for target compounds

Sulfonate derivatives have been widely studied owning to their strong affinity for lipids, which can easily cross the plant cuticle to perform their biological activities [12, 13]. Aryl sulfonate esters have been widely used for agricultural and pharmaceutical research due to their antiviral [14, 15], antibacterial [16], antifungal [17], insecticidal [18], and anticancer [19] properties. And more aryl sulfonate compounds with high efficacy in insecticidal, antibacterial, and antiviral activities can be obtained by structural modification [20, 21], such as, genite, irosustat, chlorfenson and nimrod, etc. In recent years, 1,3,4-thiadiazole derivatives and amide derivatives have become a major focus for the development of novel bactericides, insecticides, and fungicides [22–26], and a number of commercial agents containing thiadiazole/amide structures have been discovered and commercialized, such as bismerthiazol, thiodiazole copper, boscalid, diflubenzuron, and chlorantraniliprole, etc. [21]. In view of the above, a series of novel sulfonate derivatives containing 1,3,4-thiadiazole/amide backbone was designed and synthesized for further development of efficient and active lead compounds (Fig. 1) and evaluated for their biological activities. To our delight, most of the title compounds showed average biological activity against bacteria but most of the target compounds exhibited moderate to excellent insecticidal activity. The high insecticidal activity of the compounds was validated by further preliminary mechanism of action studies and molecular docking.

## Results and discussion

### Chemical synthesis

**Scheme 1:**
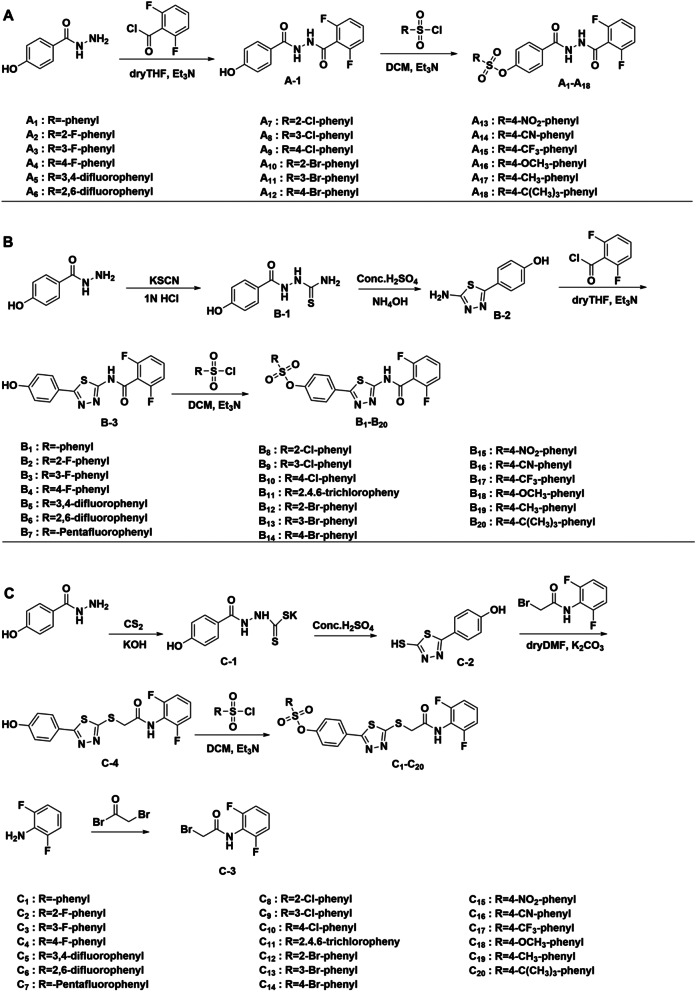
Synthetic routes for the target compounds

The synthetic route of target compounds is shown in Scheme 1. 4-hydroxybenzhydrazideproduce intermediate **A-1** by nucleophilic substitution reaction with acyl chloride, and intermediate A-1 is sulfonated with sulfonyl chloride to produce target compounds **A**_**1**_-**A**_**18**_ (Scheme 1A). 4-hydroxybenzhydrazide produce amino thiourea compounds by addition reaction with potassium thiocyanide under acidic condition, and intermediate **B-1** produce target compounds **B**_**1**_**-B**_**20**_ (Scheme 1B) by cyclization reaction. Intermediate **B-2** is formed by cyclization of intermediate **B-1**, and intermediate **B-3** is sulfonated with sulfonyl chloride to form target compounds **B**_**1**_**-B**_**20**_. The synthesis route of target compounds **C**_**1**_**-C**_**20**_ is shown in Scheme 1C. 4-hydroxybenzhydrazidereacts with carbon disulfide in a condensation reaction to form intermediate **C-1** under alkaline conditions. Intermediate **C-1** is cyclized to form intermediate **C-2**, intermediate **C-2** and **C-3** react with nucleophilic substitution to form intermediate **C-4**, intermediate **C-4** and sulfonyl chloride react with sulfonation to form the target compound **C**_**1**_**-C**_**20**_. The chemical structures of all the target compounds, **A**_**1**_**-A**_**18**_, **B**_**1**_**-B**_**20**_ and **C**_**1**_**-C**_**20**_, have been confirmed by 1^1^H NMR, 13^13^C NMR spectroscopy and high-resolution mass spectrometry. Physicochemical properties are provided in supporting information.

### Insecticidal activity

The insecticidal activities of the target compounds are shown in Table 1, where diflubenzuron is used as a positive control. It can be seen that most of the target compounds show excellent insecticidal activities against both *P. xylostella* and *Myzus persicae*. Among them, compounds **A**_**8**_, **A**_**13**_, **A**_**16**_, **B**_**1**_, **B**_**3**_, **B**_**4**_, **B**_**5**_, **B**_**10**_, **B**_**12 − 20**_, **C**_**3**_, **C**_**5**_, **C**_**9**_, **C**_**10**_, **C**_**14**_, **C**_**15**_, **C**_**17**_ and **C**_**19**_ all show 100% mortality against *P. xylostella* at a concentration of 500 *µ*g/mL, and compounds **B**_**15**_, **C**_**14**_, **C**_**15**_, **C**_**17**_ and **C**_**19**_ show insecticidal activity against the *P. xylostella* of more than 80% at 50 *µ*g/mL, notably compound **B**_**15**_ still show 100% mortality against *P. xylostella* at both 50 and 500 *µ*g/mL concentrations.

In order to assess the insecticidal potency, the compounds that showed more than 90% mortality at 50 *µ*g/mL concentration are further investigated to obtain their half lethal concentration (LC_50_). As shown in Table 2, **B**_**15**_, **C**_**14**_, **C**_**15**_, **C**_**17**_ and **C**_**19**_ show better insecticidal activity against *Plutella xylostella* with LC_50_ values ranging from 7 to 19 *µ*g/mL as compared to diflubenzuron with LC_50_ value of 24.08 *µ*g/mL. Among them, **B**_**15**_ is the compound with the highest mortality against *Plutella xylostella* with an LC_50_ value of 7.61 *µ*g/mL, which was about three times more active than that of diflubenzuron.

**Table 1:** Insecticidal activity of title compounds **A**_**1**_-**A**_**18**_, **B**_**1**_-**B**_**20**_ and **C**_**1**_-**C**_**20**_ against *P. xylostella* and *Myzus persicae*

Compd.	P. xylostella		Myzus persicae
	500 µg/mL (%) ^a^	50 µg/mL (%) ^a^	500 µg/mL (%) ^a^
**A**_**1**_	90.00 ± 0.00	73.33 ± 5.77	36.67 ± 5.77
**A**_**2**_	93.33 ± 5.77	70.00 ± 10.00	16.67 ± 5.77
**A**_**3**_	80.00 ± 10.00	33.33 ± 5.77	26.67 ± 5.77
**A**_**4**_	80.00 ± 0.00	70.00 ± 0.00	60.00 ± 10.00
**A**_**5**_	76.67 ± 5.77	30.00 ± 0.00	83.33 ± 5.77
**A**_**6**_	76.67 ± 11.55	30.00 ± 10.00	6.67 ± 5.77
**A**_**7**_	80.00 ± 0.00	23.33 ± 5.77	16.67 ± 5.77
**A**_**8**_	100	50.00 ± 10.00	20.00 ± 0.00
**A**_**9**_	93.33 ± 5.77	60.00 ± 0.00	33.33 ± 5.77
**A**_**10**_	73.33 ± 11.55	26.67 ± 11.55	40.00 ± 0.00
**A**_**11**_	86.67 ± 5.77	20.00 ± 0.00	23.33 ± 11.55
**A**_**12**_	66.67 ± 5.77	63.33 ± 5.77	26.67 ± 5.77
**A**_**13**_	100	53.33 ± 5.77	53.33 ± 5.77
**A**_**14**_	86.67 ± 5.77	53.33 ± 5.77	66.67 ± 5.77
**A**_**15**_	90.00 ± 0.00	60.00 ± 10.00	66.67 ± 11.55
**A**_**16**_	100	60.00 ± 0.00	50.00 ± 10.00
**A**_**17**_	40.00 ± 0.00	13.33 ± 5.77	23.33 ± 5.77
**A**_**18**_	93.33 ± 5.77	73.33 ± 5.77	6.67 ± 5.77
**B**_**1**_	100	76.67 ± 5.77	30.00 ± 0.00
**B**_**2**_	90.00 ± 10.00	20.00 ± 0.00	3.33 ± 5.77
**B**_**3**_	100	40.00 ± 10.00	10.00 ± 0.00
**B**_**4**_	100	50.00 ± 0.00	36.67 ± 5.77
**B**_**5**_	100	40.00 ± 0.00	86.67 ± 5.77
**B**_**6**_	86.67 ± 5.77	10.00 ± 0.00	23.33 ± 5.77
**B**_**7**_	96.67 ± 5.77	40.00 ± 10.00	26.67 ± 5.77
**B**_**8**_	93.33 ± 5.77	36.67 ± 11.55	10.00 ± 0.00
**B**_**9**_	86.67 ± 11.55	13.33 ± 5.77	46.67 ± 5.77
**B**_**10**_	100	700.00 ± 0.00	26.67 ± 5.77
**B**_**11**_	93.33 ± 5.77	26.67 ± 5.77	20.00 ± 0.00
**B**_**12**_	100	43.33 ± 5.77	13.33 ± 11.55
**B**_**13**_	100	43.33 ± 5.77	16.67 ± 5.77
**B**_**14**_	100	66.67 ± 5.77	80.00 ± 10.00
**B**_**15**_	100	100	50.00 ± 10.00
**B**_**16**_	100	70.00 ± 10.00	20.00 ± 10.00
**B**_**17**_	100	50.00 ± 0.00	30.00 ± 0.00
**B**_**18**_	100	66.67 ± 5.77	26.67 ± 5.77
**B**_**19**_	100	60.00 ± 0.00	36.67 ± 5.77
**B**_**20**_	100	46.67 ± 5.77	30.00 ± 0.00
**C**_**1**_	86.67 ± 11.55	76.67 ± 5.77	10.00 ± 0.00
**C**_**2**_	70.00 ± 10.00	6.67 ± 5.77	16.67 ± 5.77
**C**_**3**_	100	50.00 ± 10.00	13.33 ± 11.55
**C**_**4**_	93.33 ± 5.77	83.33 ± 5.77	16.67 ± 5.77
**C**_**5**_	100	53.33 ± 5.77	83.33 ± 5.77
**C**_**6**_	86.67 ± 5.77	23.33 ± 5.77	26.67 ± 5.77
**C**_**7**_	73.33 ± 11.55	30.00 ± 10.00	50.00 ± 10.00
**C**_**8**_	93.33 ± 5.77	30.00 ± 0.00	6.67 ± 5.77
**C**_**9**_	100	36.67 ± 5.77	10.00 ± 0.00
**C**_**10**_	100	70.00 ± 10.00	50.00 ± 10.00
**C**_**11**_	76.67 ± 5.77	20.00 ± 0.00	20.00 ± 0.00
**C**_**12**_	96.67 ± 5.77	36.67 ± 5.77	3.33 ± 5.77
**C**_**13**_	83.33 ± 5.77	10.00 ± 0.00	16.67 ± 5.77
**C**_**14**_	100	93.33 ± 5.77	30.00 ± 10.00
**C**_**15**_	100	90.00 ± 0.00	36.67 ± 5.77
**C**_**16**_	83.33 ± 5.77	66.67 ± 5.77	26.67 ± 5.77
**C**_**17**_	100	93.33 ± 5.77	23.33 ± 11.55
**C**_**18**_	93.33 ± 5.77	66.67 ± 5.77	33.33 ± 5.77
**C**_**19**_	100	90.00 ± 10.00	16.67 ± 5.77
**C**_**20**_	66.67 ± 5.77	33.33 ± 5.77	40.00 ± 0.00
**Diflubenzuron**	100	80.00 ± 0.00	3.00 ± 5.77

^a^All results are expressed as mean ± SD.

**Table 2:** LC_50_ values of some compounds with excellent insecticidal activity against the *P. xylostella*

Compd.	Plutella xylostella		
	Regression eq.	R^2^	LC_50_(µg/mL) ^a^
**B**_**15**_	y = 2.59x + 2.72	0.98	7.61 ± 1.62
**C**_**14**_	y = 3.23x + 0.88	0.99	18.85 ± 3.46
**C**_**15**_	y = 2.79x + 1.70	0.97	15.28 ± 2.52
**C**_**17**_	y = 3.47x + 0.70	0.97	17.38 ± 3.16
**C**_**19**_	y = 2.73x + 1.69	0.99	16.37 ± 3.41
**Diflubenzuron**	y = 2.34x + 1.77	0.99	24.08 ± 2.31

^a^All results are expressed as mean ± SD.

### Changes of chitinase, juvenile hormone and ecdysteroids in *P. xylostella*

**Fig. 2:**
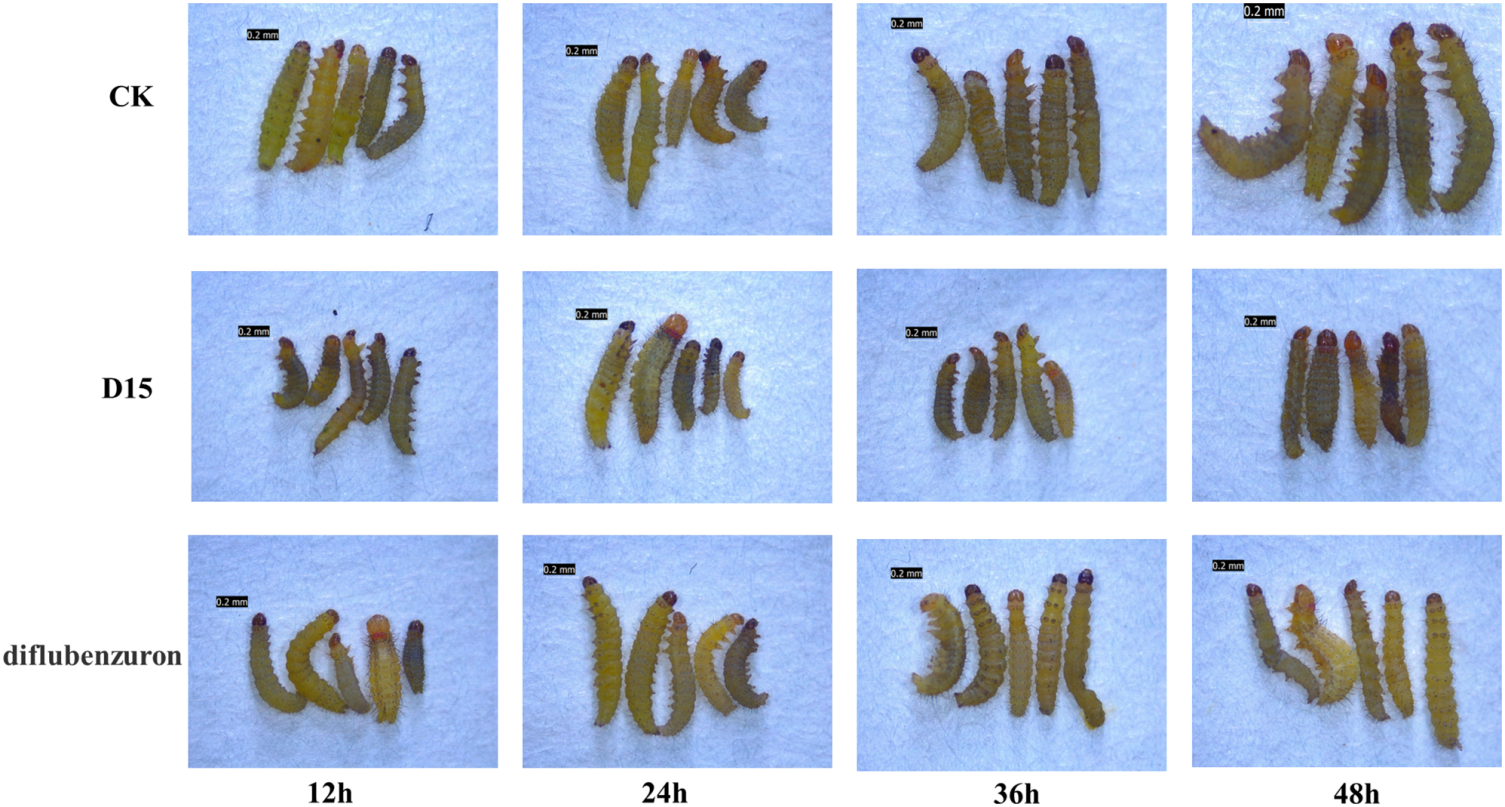
Morphological changes of the *P. xylostella* feeding on the drug-containing leaves at different time periods

**Fig. 3:**
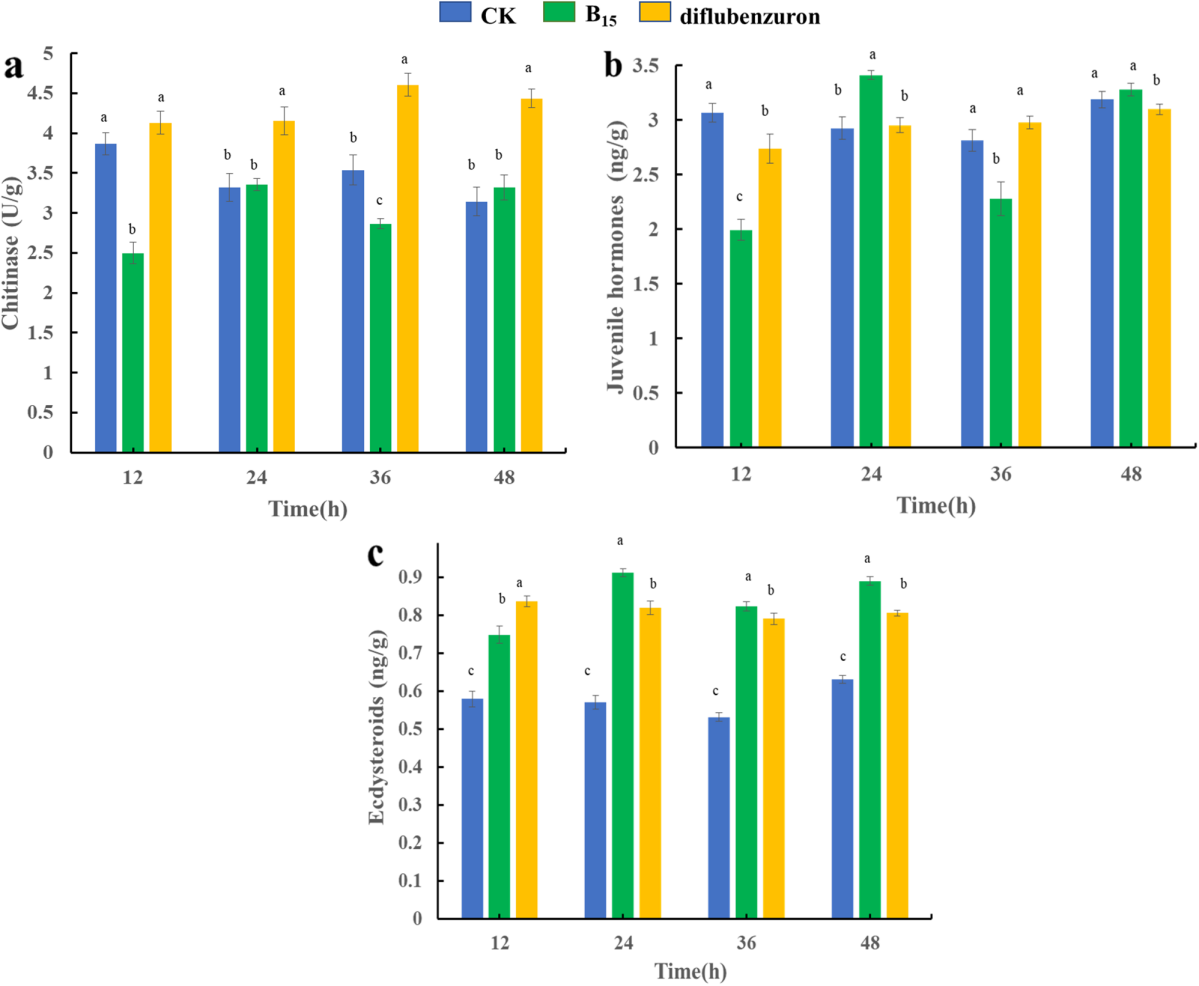
Graphical representations of changes in chitinase, juvenile hormone and ecdysteroids in the *P. xylostella* feeding on drug-containing leaves. Values are the means and standard deviations of three independent experiments. Different lowercase letters indicate values with significant differences among different treatment groups, according to one-way ANOVA (*P* < 0.05)

The growth and development of insects are mainly affected by chitinase, and the interaction of juvenile hormone and ecdysteroids also affects the activity and content of chitinase, which in turn affects the metamorphosis and development of insects. In order to further evaluate the possible insecticidal mechanism of action of compound **B**_**15**_, it firstly is observed the morphological changes of *P. xylostella* feeding on drug-containing leaves, and then tested the effects of compound **B**_**15**_ on chitinase, juvenile hormone and ecdysteroids of *P. xylostella*. It can be seen that *P. xylostella* in the treated group after feeding on the drug-containing leaves over time the death of the worms is accompanied by body stiffness, as compared to that of diflubenzuron as a positive control (Fig. 2). To further explore the possible mechanism of action of the title compound in killing the insects, we test the changes in chitinase, juvenile hormone and ecdysteroids in *P. xylostella* after feeding on the drug-containing leaves (Fig. 3). The inhibition of *P. xylostella* chitinase activity by compound **B**_**15**_ shows a tendency of increasing and then decreasing, and the inhibition of chitinase activity is most obvious at 12 h, whereas the inhibition of chitinase activity by diflubenzuron is almost non-existent (Fig. 3a). The effect of compound **B**_**15**_ on juvenile hormones appears to follow the same trend as its effect on chitinase activity (Fig. 3b). The levels of ecdysteroids are higher in both compound **B**_**15**_ and diflubenzuron-treated *P. xylostella* than in the blank control group (CK), and there is no inhibition of ecdysteroids by compound **B**_**15**_ and diflubenzuron (Fig. 3c). Chitinase plays an important role in the growth and development of insects, and this result suggests that compound **B**_**15**_ may prevent *P. xylostella* from completing the normal metamorphosis and development process by inhibiting the activity of chitinase, leading to its death.

### Molecular docking

**Fig. 4:**
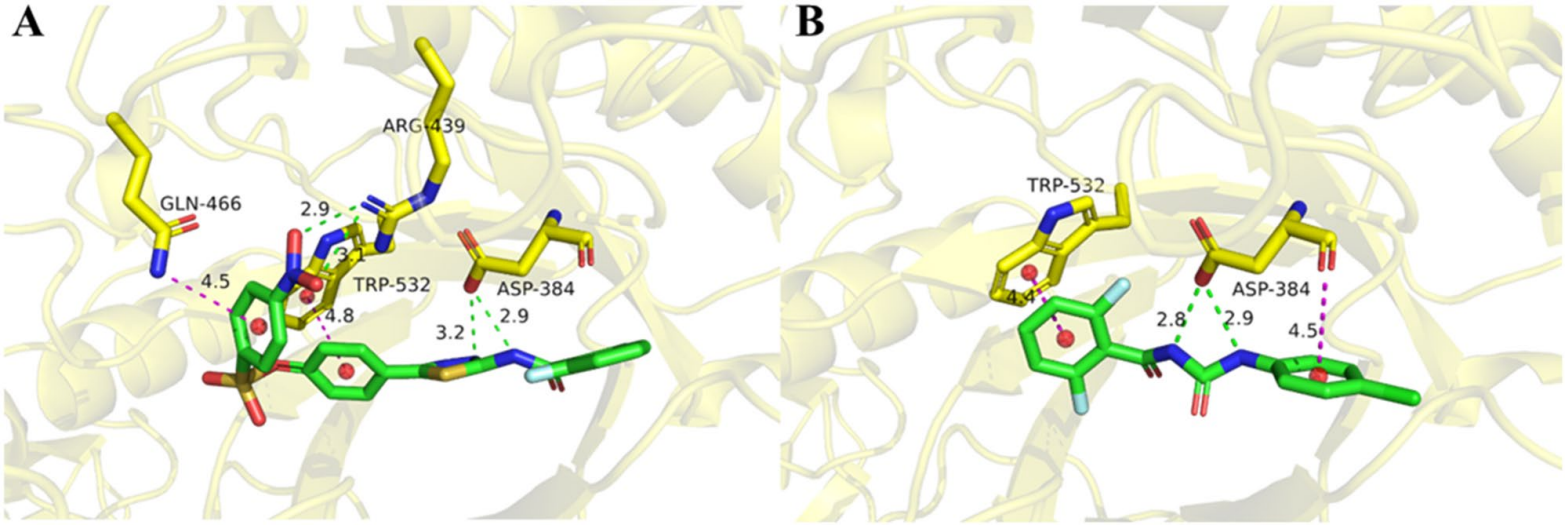
Molecular docking diagram of diflubenzuron (A) and compound **B**_**15**_ (B)

Chitin is essential in insect growth and development, and it has been shown that the title compound disrupts chitinase activity, which in turn may cause dysregulation of chitin synthesis and catabolism in insects; therefore, the chitinase-related protein (PDB ID: 6JMA) has been selected to be used for molecular docking. In order to verify the possible insecticidal mechanism of the title compound **B**_**15**_, it is docked at the theoretical binding site of the chitinase protein, diflubenzuron is used as a positive control. The interactions of **B**_**15**_ and diflubenzuron with the chitinase protein are shown in Fig. 4. In the docking complex of diflubenzuron and chitinase protein (Fig. 4A) the two *N* atoms of the amide bond form two hydrogen bonds (2.8 Å and 2.9 Å) with the key residue ASP384, which is essential for the binding of the inhibitor and chitinase protein. The benzene ring forms a π-π stacking interaction with residues TRP532 and ASP384 (4.4 Å and 4.5 Å). As shown in Fig. 4B, the thiazole ring and amide bond of **B**_**15**_ forms two hydrogen bonds (3.2 Å and 2.9 Å) with ASP384 as well as the nitro group on the benzene ring with ARG239 (2.9 Å and 3.1 Å), which is two more than that of the diflubenzuron. The benzene ring forms a π-π stacking interaction with ARG439 and GLN466 (4.8 Å and 4.5 Å), respectively. All these differences explain the more active insecticidal activity of **B**_**15**_ against the *P. xylostella* than that of diflubenzuron.

### *In vitro* antibacterial activity

In order to explore the effect of the synthesized target compounds beyond insecticides, antibacterial activity experiments are carried out. The antibacterial activities of the title compounds in vitro are shown in Table 3. Most of the compounds show moderate inhibitory activity against the tested bacteria. Among them, some of the compounds show a good inhibitory activity against *Xoo* and *Xoc*, and compounds **A**_**4**_ and **C**_**4**_ both showed more than 90% inhibitory activity against *Xoo* at a concentration of 100 *µ*g/mL. Then, we test the EC_50_ values of those title compounds that showed better activity against *Xoo* and *Xoc*, the EC_50_ values of antibacterial activity of compound **A**_**4**_ and compound **C**_**4**_ against *Xoo* are 24.87 and 30.04 *µ*g/mL, respectively. The EC_50_ values of compounds **A**_**4**_, **A**_**15**_, **A**_**16**_, **B**_**7**_, **C**_**4**_ and **C**_**7**_ of that inhibitory activity against *Xoc* are 30.12, 39.59, 18.75, 67.9, 43.85 and 54.56 *µ*g/mL, respectively (Table 4).

**Table 3:** *In vitro* antibacterial activity results of the title compounds

Compd.	Xoo		Xoc		Xac		Psa	
	100 µg/mL(%)^a^	50 µg/mL(%)^a^	100 µg/mL(%)^a^	50 µg/mL(%)^a^	100 µg/mL(%)^a^	50 µg/mL(%)^a^	100 µg/mL(%)^a^	50 µg/mL(%)^a^
**A**_**1**_	18.56 ± 1.33	21.11 ± 1.15	70.33 ± 2.53	36.20 ± 1.10	35.03 ± 1.23	26.05 ± 1.03	27.91 ± 3.15	12.88 ± 1.51
**A**_**2**_	33.16 ± 0.93	22.78 ± 0.76	53.43 ± 1.81	27.27 ± 2.09	46.87 ± 2.78	23.03 ± 1.14	21.12 ± 2.52	11.64 ± 0.99
**A**_**3**_	41.16 ± 1.21	36.85 ± 1.35	41.63 ± 2.19	26.16 ± 1.20	46.03 ± 1.24	20.18 ± 2.48	18.52 ± 2.46	10.43 ± 1.44
**A**_**4**_	90.59 ± 3.32	39.67 ± 1.45	66.67 ± 1.54	40.19 ± 1.27	39.00 ± 2.34	23.96 ± 3.30	22.37 ± 2.01	12.62 ± 2.05
**A**_**5**_	42.74 ± 1.47	10.73 ± 1.00	78.15 ± 1.54	45.45 ± 1.44	49.00 ± 4.50	25.08 ± 1.32	12.94 ± 2.54	8.12 ± 2.06
**A**_**6**_	13.46 ± 1.15	33.51 ± 1.58	66.99 ± 1.66	40.19 ± 1.66	56.64 ± 1.34	34.49 ± 1.03	10.16 ± 1.45	8.69 ± 4.39
**A**_**7**_	39.67 ± 1.85	58.58 ± 1.40	56.94 ± 1.27	36.20 ± 4.45	42.63 ± 0.94	28.86 ± 3.40	20.10 ± 1.46	8.31 ± 2.45
**A**_**8**_	13.28 ± 1.30	38.79 ± 2.06	69.06 ± 1.10	49.76 ± 0.83	52.54 ± 3.02	25.55 ± 1.03	16.06 ± 2.20	9.75 ± 1.42
**A**_**9**_	11.79 ± 1.52	65.00 ± 2.71	60.29 ± 1.27	37.16 ± 1.20	42.44 ± 2.37	27.06 ± 4.71	15.07 ± 2.86	6.61 ± 2.08
**A**_**10**_	68.78 ± 3.86	20.99 ± 3.56	45.45 ± 2.91	24.72 ± 1.68	35.08 ± 1.36	28.68 ± 1.53	9.53 ± 0.34	5.63 ± 2.04
**A**_**11**_	21.78 ± 1.76	23.17 ± 1.05	61.08 ± 1.46	41.47 ± 2.92	34.89 ± 2.14	26.85 ± 2.92	14.78 ± 4.83	5.52 ± 1.21
**A**_**12**_	64.69 ± 1.19	52.67 ± 1.10	56.78 ± 2.46	33.97 ± 2.53	30.08 ± 1.67	24.97 ± 3.74	9.79 ± 1.69	5.29 ± 2.59
**A**_**13**_	31.35 ± 0.50	32.41 ± 1.92	35.41 ± 2.09	23.13 ± 1.00	36.45 ± 2.92	19.03 ± 1.13	20.28 ± 2.92	11.75 ± 3.73
**A**_**14**_	17.89 ± 1.94	61.91 ± 1.14	85.96 ± 1.68	37.00 ± 1.46	32.39 ± 1.82	24.50 ± 4.05	10.23 ± 1.69	6.31 ± 3.95
**A**_**15**_	15.58 ± 2.17	54.32 ± 2.61	94.74 ± 0.48	72.41 ± 1.00	40.84 ± 3.92	25.73 ± 1.49	13.24 ± 3.88	5.18 ± 4.77
**A**_**16**_	63.76 ± 2.72	25.74 ± 4.09	100	84.69 ± 2.91	31.26 ± 2.30	28.04 ± 2.64	10.01 ± 2.54	5.70 ± 3.51
**A**_**17**_	10.96 ± 0.60	34.26 ± 4.29	49.92 ± 2.64	33.33 ± 1.93	49.71 ± 1.96	27.60 ± 1.64	0.55 ± 2.92	0.91 ± 2.61
**A**_**18**_	11.49 ± 0.59	27.92 ± 3.26	55.50 ± 4.78	45.30 ± 1.00	42.16 ± 0.90	19.39 ± 1.58	2.86 ± 0.61	0
**B**_**1**_	68.78 ± 3.86	25.15 ± 4.24	48.00 ± 0.35	32.67 ± 1.33	60.79 ± 2.65	40.40 ± 1.25	9.29 ± 0.71	5.47 ± 1.03
**B**_**2**_	20.99 ± 3.56	8.07 ± 4.66	38.00 ± 0.53	25.80 ± 0.20	49.56 ± 2.50	35.68 ± 1.25	14.25 ± 2.15	7.70 ± 1.28
**B**_**3**_	44.22 ± 2.02	5.13 ± 2.52	35.07 ± 0.12	29.67 ± 0.12	66.22 ± 3.18	43.71 ± 1.43	15.14 ± 1.53	6.32 ± 1.04
**B**_**4**_	23.30 ± 1.02	0	42.07 ± 0.42	27.47 ± 0.81	43.52 ± 2.41	30.81 ± 1.03	2.89 ± 2.15	2.98 ± 0.46
**B**_**5**_	21.78 ± 1.76	0	41.53 ± 0.31	22.33 ± 0.31	69.24 ± 1.73	40.18 ± 1.47	5.97 ± 4.45	1.38 ± 2.15
**B**_**6**_	23.17 ± 1.05	11.18 ± 4.12	41.53 ± 0.90	31.40 ± 1.40	58.34 ± 2.20	35.71 ± 4.68	29.9 ± 1.23	14.84 ± 1.39
**B**_**7**_	64.69 ± 1.19	39.33 ± 0.34	65.33 ± 0.31	33.73 ± 1.15	47.58 ± 2.67	30.77 ± 1.04	9.02 ± 4.04	6.84 ± 1.84
**B**_**8**_	52.67 ± 1.10	8.78 ± 0.81	39.40 ± 1.06	28.93 ± 0.83	49.19 ± 2.94	26.92 ± 2.54	27.09 ± 1.09	10.6 ± 1.34
**B**_**9**_	31.35 ± 0.50	8.24 ± 1.18	41.93 ± 0.31	29.80 ± 0.35	15.36 ± 2.22	34.52 ± 3.95	6.48 ± 0.71	2.98 ± 2.84
**B**_**10**_	32.41 ± 1.92	7.91 ± 2.08	30.27 ± 0.81	24.80 ± 0.20	47.49 ± 1.39	39.39 ± 1.27	4.10 ± 4.52	0
**B**_**11**_	17.89 ± 1.94	12.11 ± 0.75	43.80 ± 0.72	31.13 ± 0.42	31.02 ± 0.99	22.41 ± 1.59	2.50 ± 3.73	0
**B**_**12**_	61.91 ± 1.14	13.26 ± 1.43	38.73 ± 0.76	21.27 ± 0.12	15.78 ± 1.86	4.86 ± 1.59	3.98 ± 1.84	0
**B**_**13**_	15.58 ± 2.17	21.88 ± 1.52	35.13 ± 0.76	29.00 ± 1.06	35.27 ± 2.52	24.04 ± 1.78	2.58 ± 1.95	0
**B**_**14**_	54.32 ± 2.61	13.91 ± 0.98	41.60 ± 0.35	33.00 ± 0.20	30.36 ± 2.62	20.07 ± 2.31	0	0
**B**_**15**_	63.76 ± 2.72	15.28 ± 0.81	44.40 ± 1.40	28.93 ± 0.31	45.55 ± 2.37	39.24 ± 1.03	0	0
**B**_**16**_	25.74 ± 4.09	13.48 ± 1.16	41.00 ± 1.25	18.27 ± 0.23	37.25 ± 1.28	30.45 ± 3.00	0	0
**B**_**17**_	10.96 ± 0.60	0	52.20 ± 1.11	37.60 ± 0.40	58.43 ± 1.20	32.72 ± 1.08	0	0
**B**_**18**_	34.26 ± 4.29	14.13 ± 2.54	47.73 ± 0.31	27.93 ± 0.12	63.10 ± 2.92	35.60 ± 2.60	11.94 ± 2.23	8.00 ± 2.09
**B**_**19**_	11.49 ± 0.59	3.82 ± 0.41	53.00 ± 0.35	39.47 ± 0.61	63.67 ± 2.89	32.86 ± 1.03	5.27 ± 2.65	4.69 ± 2.96
**B**_**20**_	27.92 ± 3.26	6.87 ± 0.59	38.27 ± 0.61	23.60 ± 0.20	47.77 ± 1.81	22.27 ± 2.07	5.89 ± 2.48	3.87 ± 1.63
**C**_**1**_	65.43 ± 0.49	35.33 ± 1.47	58.62 ± 1.39	31.90 ± 0.74	52.52 ± 1.45	41.19 ± 1.43	38.96 ± 1.27	22.22 ± 1.75
**C**_**2**_	55.81 ± 2.43	43.24 ± 1.15	39.60 ± 1.25	18.00 ± 0.54	49.85 ± 1.05	38.96 ± 1.61	41.39 ± 2.37	24.72 ± 2.40
**C**_**3**_	64.67 ± 1.57	26.86 ± 0.57	34.56 ± 1.24	25.77 ± 1.87	62.74 ± 1.22	49.04 ± 1.22	29.65 ± 1.15	11.11 ± 0.98
**C**_**4**_	92.10 ± 1.29	71.62 ± 0.16	87.93 ± 0.54	51.19 ± 1.25	64.96 ± 1.00	46.07 ± 2.23	32.43 ± 3.23	16.11 ± 1.15
**C**_**5**_	42.48 ± 2.31	26.00 ± 1.48	41.24 ± 0.47	24.06 ± 1.84	49.33 ± 2.26	36.52 ± 1.41	26.94 ± 2.41	11.67 ± 1.91
**C**_**6**_	59.62 ± 1.44	29.43 ± 1.74	38.24 ± 2.28	26.52 ± 1.44	43.41 ± 1.34	38.67 ± 1.33	11.88 ± 1.50	0
**C**_**7**_	82.19 ± 1.44	47.71 ± 0.86	84.12 ± 1.84	41.04 ± 0.31	62.89 ± 2.04	46.37 ± 2.06	52.22 ± 1.82	34.44 ± 0.94
**C**_**8**_	57.81 ± 1.08	31.05 ± 3.00	36.33 ± 1.99	20.45 ± 0.54	58.44 ± 1.02	34.81 ± 1.70	26.81 ± 1.88	10.56 ± 0.94
**C**_**9**_	56.48 ± 1.90	28.76 ± 1.44	42.40 ± 2.37	34.08 ± 1.03	62.30 ± 2.27	52.15 ± 1.12	38.19 ± 1.26	27.57 ± 1.39
**C**_**10**_	66.19 ± 1.90	38.19 ± 1.44	48.53 ± 2.37	33.06 ± 1.30	58.15 ± 1.48	49.93 ± 1.12	25.97 ± 1.07	15.63 ± 0.95
**C**_**11**_	59.14 ± 1.78	32.29 ± 1.74	51.74 ± 1.14	28.02 ± 0.71	56.07 ± 2.58	48.74 ± 1.61	11.94 ± 1.54	3.96 ± 0.95
**C**_**12**_	61.43 ± 1.31	40.29 ± 0.57	30.88 ± 0.54	25.97 ± 1.34	57.56 ± 2.12	37.26 ± 1.48	17.29 ± 2.32	1.67 ± 1.91
**C**_**13**_	68.19 ± 1.67	40.57 ± 1.25	32.58 ± 1.03	22.22 ± 1.79	53.56 ± 1.02	39.63 ± 2.02	1.53 ± 1.70	1.18 ± 1.03
**C**_**14**_	55.33 ± 1.00	34.38 ± 3.08	46.35 ± 1.39	34.01 ± 2.46	44.15 ± 3.70	36.44 ± 2.31	39.86 ± 2.23	24.51 ± 1.22
**C**_**15**_	69.81 ± 3.57	31.71 ± 0.49	23.38 ± 1.93	14.45 ± 1.18	55.41 ± 2.57	44.52 ± 2.25	46.04 ± 2.76	37.43 ± 1.92
**C**_**16**_	54.00 ± 1.31	24.76 ± 1.47	38.17 ± 1.39	20.04 ± 1.14	54.15 ± 3.03	44.81 ± 3.03	35.76 ± 1.67	11.67 ± 1.85
**C**_**17**_	55.33 ± 1.00	35.52 ± 1.29	70.28 ± 1.55	41.24 ± 1.13	48.59 ± 2.06	45.85 ± 1.03	9.65 ± 1.70	1.74 ± 1.59
**C**_**18**_	63.62 ± 1.94	36.86 ± 0.49	47.99 ± 0.83	26.58 ± 1.02	53.63 ± 1.81	43.48 ± 2.45	2.71 ± 2.18	0
**C**_**19**_	62.19 ± 0.87	36.19 ± 0.72	76.35 ± 1.70	43.08 ± 1.05	54.74 ± 0.68	44.52 ± 3.34	7.78 ± 1.89	3.61 ± 1.48
**C**_**20**_	74.00 ± 1.31	39.43 ± 1.87	29.65 ± 1.06	18.13 ± 0.92	50.44 ± 1.02	39.26 ± 2.63	7.36 ± 1.03	0
**BT**	100	77.00 ± 1.46	84.00 ± 2.36	42.00 ± 2.13	57.00 ± 1.18	41.00 ± 0.64	/	/
**TZ**	/	/	/	/	97.00 ± 2.64	40.00 ± 1.70	60.00 ± 3.24	42.00 ± 1.13
**TC**	100	54.00 ± 1.12	62.00 ± 2.48	33.00 ± 2.41	40.00 ± 0.57	27.00 ± 2.32	37.00 ± 2.80	22.00 ± 1.70

^a^All results are expressed as mean ± SD.

**Table 4:** EC_50_ values of some compounds with better inhibitory activity against *Xoo*, *Xoc*

Compd.	Xoo				Xoc			
	Regression eq.	R^2^	EC_50_(µg/mL)^a^			Regression eq.	R^2^	EC_50_(µg/mL)^a^
**A**_**4**_	y = 1.83x + 2.44	0.96	24.87 ± 1.22			y = 2.15x + 1.81	0.95	30.12 ± 1.03
**A**_**15**_	-	-	-			y = 4.19x-1.69	0.99	39.59 ± 1.44
**A**_**16**_	-	-	-			y = 2.16x + 2.25	0.95	18.75 ± 1.16
**B**_**7**_	y = 1.27x + 2.69	0.96	66.88 ± 2.23			y = 2.42x + 0.57	0.97	67.90 ± 1.90
**C**_**4**_	y = 1.58x + 2.66	0.97	30.04 ± 2.01			y = 3.15x-0.17	0.99	43.85 ± 2.28
**C**_**7**_	-	-	-			y = 3.90x-1.78	0.99	54.56 ± 1.58
**BT**	y = 2.21x + 1.91	0.99	25.12 ± 1.03			y = 1.39x + 2.38	0.98	76.38 ± 3.23
**TC**	y = 1.41x + 2.66	0.99	45.81 ± 1.72			y = 1.50x + 1.82	0.94	130.49 ± 2.50

^a^All results are expressed as mean ± SD.

## Conclusion

In summary, 58 novel series of sulfonate derivatives containing an amide backbone were designed and synthesized. They have been evaluated for their antibacterial and insecticidal activities. The results of the bioactivity tests indicated that the compounds show fair antibacterial activity, but most of them showed good insecticidal activity against the *P. xylostella*. Among them, compound **B**_**15**_ showed the best insecticidal activity against *P. xylostella* with LC_50_ of 7.61 *µ*g/mL, which is much lower than the control agent diflubenzuron. Further preliminary studies on the insecticidal mechanism indicated that compound **B**_**15**_ may exert its insecticidal activity by inhibiting chitinase and juvenile preserving hormone in the body of the *P. xylostella*, and this result was also consistent with the molecular docking experiments. Therefore, compound **B**_**15**_ may be a promising insecticide candidate.

## Materials and methods

### Equipment and materials

Melting points of new compounds were determined on an X-4 digital display micro melting point apparatus (Henan, Gongyi Yuhua Instrument Co., Ltd, China) and were uncorrected. The NMR spectra of the title compounds were obtained on a JEOL-ECX500 apparatus (JEOL, Japan) or a Bruker Biospin AG-400 apparatus (Bruker Corporation, Germany) using tetramethylsilane as the internal standard. High-resolution mass spectra were recorded with a Thermo Scientific UltiMate 3000 spectrometer (Thermo Fisher Scientific, USA). Optical density was recorded on a Cytation™ 5 multi-mode readers (BioTek Instruments, USA). All chemical reagents were obtained from commercial suppliers. All strains used (*Xoo*, *Xac*, *Psa* and *Xoc*) and the test insects (*Plutella xylostella* and *Myzus persicae*) were provided by the laboratory of Guizhou University. All ELISA kits were obtained from commercial suppliers (Jiangsu Meimian Industrial Co., Ltd, China).

### Preparation procedure of intermediate compounds

Synthesis of intermediate **A-1**: following the method described in the literature [27], 4-hydroxybenzhydrazide (65.7 mmol) and Et_3_N (131.4 mmol) were added to a clean 250 mL three-necked flask with tetrahydrofuran (130 mL), at 25 ℃. Then 2,6-difluorobenzoyl chloride (65.7 mmol) was added to the mixture at ice bath. The reaction would be accomplished quickly in 5 min (monitored by TLC). Then the reaction mixture was quenched by adding 100 mL H_2_O and extracted with ethyl acetate (100mL × 3). The collected organic phase was extracted with NaOH solution and the water phase was adjusted to pH = 4–5 with diluted hydrochloric acid. Then ethyl acetate was added to extract and concentrate to get the crucial solid, which was then recrystallized with anhydrous ethanol to obtain white crystal intermediate **A-1** (yield, 76%).

Synthesis of intermediate **B-1**: following the method described in the literature [28], 4-hydroxybenzhydrazide (65.7 mmol) was added into a clean 250 mL three-necked flask with 120 mL of 1 M HCl solution at room temperature. After stirring for 15 min, all the solid was dissolved. Then potassium thiocyanate (131.4 mmol) was added and the mixture was heated at 100 °C for 8 h until the reaction completion as monitored by TLC. The system was cooled to room temperature and a large amount of white solid appeared. The solid product was filtered and washed by water for three times and ethanol once, and then recrystallized in anhydrous ethanol to obtain intermediate **B-1**, with yield of 57.6%.

Synthesis of intermediate **B-2**: following the method in the literature [28], intermediate **B-1** (28.4 mmol) was added into a clean 50 mL three-neck flask at 25 ℃ followed with H_2_SO_4_ (20 mL) while stirring. After 6 h the reaction was completed. Then the reaction mixture was slowly poured into crushed ice, and 25% ammonia water was added to adjust the solution to pH = 8–10 during stirring and a large amount of white solid precipitated. The precipitate was filtered and washed with water three times and dried. The solid was then recrystallized in anhydrous ethanol to obtain intermediate **B-2**, with yield of 32.8%.

Synthesis of intermediate **B-3**: following the method in the literature [29],intermediate **B-2** (9.3 mmol) and Et_3_N (18.6 mmol) were added to 20 mL tetrahydrofuran in a clean 50 mL three-neck flask and stirred for 15 min at 25 ℃. Next in an ice bath, 2,6-difluorobenzoyl chloride (9.3 mmol) was slowly added and then and the mixture was stirred at room temperature till reaction was completed according to TLC. Then the reaction mixture was quenched by adding 20 mL H_2_O and extracted with ethyl acetate (20 mL× 3). The collected organic phase was extracted with NaOH solution, and the water phase was adjusted to pH = 4–5 with diluted hydrochloric acid. Then ethyl acetate was added to extract and concentrate to get the crucial solid, which was then recrystallized with anhydrous ethanol to obtain intermediate **B-3**, with yield of 61.4%.

Synthesis of intermediate **C-1**: following the published methods [28], 4-hydroxybenzhydrazide (32.8 mmol), KOH (49.2 mmol), and 80 mL of ethanol were added sequentially to a 150 mL three-neck flask at room temperature. The solids were dissolved after 10 min stirring. The CS_2_ (49.2 mmol) was slowly added into the mixture in an ice bath. The mixture was stirred overnight at room temperature. The reaction solution was becoming, and till the reaction completed as indicated by TLC. Solid products appeared in the solution, filtered and washed by ethanol to obtain intermediate **C-1**, with yield 92%. The intermediate **C-1** was subjected directly to the next reaction without recrystallization.

Synthesis of Intermediate **C-2**: following the published methods [28], intermediate **C-1** (7.5 mmol) was slowly added to the ice-cold H_2_SO_4_ (15 mL) in 50 mL three-neck flask while stirring. The reaction was kept stirring for 4 h at an ice-water bath and poured into crushed ice. The newly appeared precipitate product was filtered to dissolve in a 20% NaOH aqueous solution. After insoluble substances were excluded, and the solution was acidified with diluted hydrochloric acid and got a precipitate, which was then filtered, washed by water and dried to obtain intermediate **C-2**, with a yield of 76.1%.

Synthesis of intermediate **C-3**: following the method described in the literature [30], 2,6-difluoroaniline (38.7 mmol) was added into 80 mL of 1,4-dioxanein a 150 mL three-necked flask and dissolved after stirring for 10 min. Then bromoacetyl bromid (50.3 mmol) early dissolved in dry dioxane (15 mL), was added dropwise to the above solution at 0 ℃ then mixture was stirred at room temperature for 5 h. Finally, the reaction mixture was poured into cold water, precipitate appeared, which was then filtered, washed by water and dried to obtain intermediate C-3, with a yield of 64%.

Synthesis of intermediate **C-4**: as reported [31], a mixture of thiazole intermediate **C-2** (15.6 mmol), anhydrous potassium carbonate (23.4 mmol), and the corresponding amide intermediate **C-3** (17.2 mmol) was added to dimethylformamide (80 mL) in a 150 mL three-necked flask at 25 ℃. The mixture was stirred for 4 h (monitored by TLC). Then, the reaction solution was poured into 80 mL cold water, and got precipitates, which was filtered, washed by water and dried to (intermediate **C-4**) was obtained, with a yield of 67.1%.

### General procedure of target compound A_1_-A_18,_ B_1_-B_20,_ C_1_-C_20_

For all target compounds, the final synthetic procedure was an esterification between the substituted benzene sulfonyl chloride and **A-1**, **B-3** or **C-4** respectively. And the reaction condition was completely same. Here, compound A series was taken for example to illustrate the reaction procedure. Intermediate **A-1** (1.7 mmol) and triethylamine (5.1 mmol) were added and dissolved in 20 mL of dichloromethane in a clean 50 mL three-necked flask at room temperature. To the flask, different substituted benzene sulfonyl chloride (2.1 mmol) was added accordingly. The mixture was stirred overnight till the reaction was completed (monitored by TLC). The reaction was quenched with 20 mL water. Then ethyl acetate was added to extract and concentrate to get the crude solid, which was purified by the column chromatography (dichloromethane: methanol = 50:1, v/v) and obtained the title compounds **A**_**1**_**-A**_**18**_ with a yield in the range of 70-96%. For title compound **B**_**1**_**-B**_**20**,_ the yield ranged 74-90% and C_1_-C_20_ 68-95%.

### Insecticidal assay

The insecticidal activity of compounds against *P. xylostella* and *Myzus persicae* was evaluated according to our previously reported methods [21]. After soaked in a solution of 500 and 50 *µ*g/mL of compounds **A**_**1**_**-A**_**18**_, **B**_**1**_**-B**_**20**_ and **C**_**1**_**-C**_**20**_, diflubenzuron (positive control), or no compound (blank control) for 10s, the fresh cabbage discs (6 cm in diameter) were air-dried and placed in 9 cm petri dishes lined with wet filter paper. Ten *P. xylostella/Myzus persicae* (third instar larvae) were placed in the petri dishes and the survival insect individual was recorded after 72 h. The mortality rate was determined by the number of survival larvae comparing to the initial number. Compounds that were active at this concentration were further tested at 5 lower gradient concentrations (6.25, 12.5, 25.00, 50.00 and 100.00 *µ*g/mL) to get LC_50_. Each treatment was repeated three times. The data were analyzed with probit analysis to obtain LC_50_ values All experiments were conducted at 25 °C using laboratory reared test insects and were repeated according to statistical requirements. The death rate was estimated by calculating the ratio of the number of insects killed to the initial number.

The adjusted mortality rate (%) is as follows:

Adjusted mortality rate (%) = (death rate in the treatment group - death rate in the blank control group)/(1 ‐ death rate in the blank control group) × 100.

### **Effect of highly active compound on chitinase, juvenile hormone and ecdysterorids in*P. xylostella***

Fresh cabbage discs (6 cm in diameter) were first soaked in a solution of 50*µ*g/mL of highly active compound for 10 s, air-dried and placed in a 9 cm petri dish lined with wet filter paper. Ten third instar larvae of *P. xylostella* were placed in the petri dishes and samples were taken and stored at − 80 °C after 12, 24, 36 and 48 h of feeding by *P. xylostella*. Chitinase, juvenile hormone, and ecdysterorids activities were determined by MicroplateReader using commercial ELISA kits following the manufacturer’s instructions. Each experiment was repeated three times. The blank control group was treated with distilled water containing 0 *µ*g/mL of the compounds, and the positive control was selected as diflubenzuron that is a commercially available insecticide.

### Molecular docking

A molecular docking station was built using the Ledock program according to the literature [32], the crystal structure of chitinase (PDB: 6JMA) of the *P. xylostella* was downloaded from the Protein Data Bank (https://www.pdb.org, accessed on 10 February 2023) and was processed with Pymol [33]. The molecular structures of highly active compound and diflubenzuron were drawn using ChemBioDraw Ultra 14.0 software and were optimized to minimize energy. A 17.5 × 15.3 × 14.7 docking box was generated with the Carboxin Standard in the protein as the center, and the docking station generated 20 ligand conformations with an RMSD less than 1.0 Å. The docking results were visualized in 3D by the Pymol software v.2.4.0.

### Antibacterial activity assay

According to previously reported procedures [21], the title compounds **A**_**1**_**-A**_**18**_, **B**_**1**_**-B**_**20**_, **C**_**1**_**-C**_**20**_ were tested in vitro against *Xoo*, *Xoc*, *Xac* and *Psa* at concentrations of 100 and 50 *µ*g/mL respectively. The commercial fungicides Bismerthiazol (BT), Zinc Thiazole (TZ) and Thiodiazole copper (TC) were selected as positive controls. The solution containing no compound was set as negative control (CK). Compounds that were active at this concentration were further tested at 5 lower gradient concentrations (6.25, 12.50, 25.00, 50.00 and 100.00 *µ*g/mL) to get EC_50_. Each treatment was repeated three times. The inhibitory effect of compounds on bacteria was calculated based on optical density values using the following formula.

I (%) = (CK-T)/T × 100.

I (%) denotes the inhibition rate. CK denotes the OD value of the treatment with solvent with no tested compound. T denotes the OD value of the treatment in tested compound group.

### Statistical analyses

All trials were performed in triplicate, and the results of each trial are shown as mean ± standard deviation (SD). Analyses of variance (ANOVAs) were performed using SPSS Statistics 24.0 with and without the assumption of equal variance (with *P* > 0.05 taken as the significance level for both). Significant differences between treatment groups are indicated using various lowercase letters.

### Electronic supplementary material

Below is the link to the electronic supplementary material.

Supplementary Material 1

## Data Availability

Data is provided within the manuscript or supplementary information files.

## Abbreviations

*Xoo*: *Xanthomonas oryzae* pv. *oryzae*
*Xoc*: *Xanthomonas oryzae* pv. *oryzicola*
*Xac*: *Xanthomonas axonopodis* pv. *citri*
*Psa*: *Pseudomonas syringae* pv. *actinidiae*
*P. xylostella*: *Plutella xylostella*
LC_50_: median lethal concentration
EC_50_: median effect concentration
TLC: thin-layer chromatography
BT: bismerthiazol
TZ: zinc thiazole
TC: thiodiazole copper
OD: optical density
ELISA: enzyme-linked immunosorbent assay
PDB: protein data bank
