# Supplementary material for: Sulfonate derivatives bearing an amide unit: design, synthesis and biological activity studies
Source: BMC Chem. 2024 Mar 6;18(1):46. doi: 10.1186/s13065-024-01151-0 (PMC10919044; doi:10.1186/s13065-024-01151-0)
Supplement: Supplementary file 1 — Supplementary Material 1 [file 13065_2024_1151_MOESM1_ESM.docx]

**Supporting Information**

**Sulfonate derivatives bearing an amide unit: design, synthesis and biological activity studies**

**You-hua Liu^1#^, Chang-kun Li^1#^, Mao-yu Nie^1^, Fa-li Wang^1^, Xiao-li Ren^1^, Lin-hong Jin^1^, Xia Zhou^1,^ ***

^#^ These authors contributed equally to this work.

^1^*National Key Laboratory of Green Pesticide, Key Laboratory of Green Pesticide and Agricultural Bioengineering, Ministry of Education, Center for R&D of Fine Chemicals of Guizhou University, Guiyang 550025, China*

*Correspondence: xzhou@gzu.edu.cn; Tel.: +86-851-3620-521

***Contents***

Characterization data of the intermediates and target compounds ………………2-24

Original spectral files of the intermediates and target compounds……………25-115


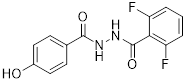
**2,6-difluoro-N'-(4-hydroxybenzoyl)benzohydrazide(中间体A-1)**:White solid, m.p. 145-147 ℃; Yield: 76%. ^1^H NMR (400 MHz, DMSO) *δ* 10.61 (s, 1H, -NH-), 10.44 (s, 1H, -NH-), 10.16 (s, 1H, -OH), 7.82 (d, *J* = 8.6 Hz, 2H, Ph-H), 7.77 – 7.51 (m, 1H, Ph-H ), 7.22 (t, *J* = 8.0 Hz, 2H Ph-H), 6.85 (d, *J* = 8.6 Hz, 2H Ph-H). ^13^C NMR (100 MHz, DMSO) *δ* 165.03, 160.83, 160.45 (d, *J* = 7.8 Hz), 159.31, 157.97 (d, *J* = 7.7 Hz), 132.47 (t, *J* = 10.1 Hz), 129.68, 122.92, 115.11, 113.59 (t, *J* = 22.6 Hz), 112.24, 112.00. HRMS (ESI) calcd for C_14_H_11_O_3_N_2_F_2_ [M＋H]^＋^: 293.0932, found 293.0727.


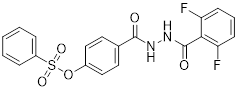
**4-(2-(2,6-difluorobenzoyl)hydrazine-1-carbonyl)phenyl-benzenesulfonate(A_1_)**: White solid, m.p.184-186 ℃; Yield: 72%. ^1^H NMR (400 MHz, DMSO) *δ* 10.77 (d, *J* = 8.0 Hz, 2H, -NH-NH-), 7.95 – 7.82 (m, 5H, Ph-H), 7.72 – 7.66 (m, 2H, Ph-H), 7.63 – 7.54 (m, 1H, Ph-H), 7.27 – 7.17 (m, 4H, Ph-H). ^13^C NMR (100 MHz, DMSO) *δ* 164.12, 160.39 (d, *J* = 7.7 Hz), 159.17, 157.91 (d, *J* = 7.6 Hz), 151.37, 135.35, 133.99, 132.59 (t, *J* = 9.9 Hz), 131.32, 130.01, 129.65, 128.35, 122.34, 113.31 (t, *J* = 22.5 Hz), 112.25, 112.01. HRMS (ESI) calcd for C_20_H_15_O_5_N_2_F_2_S [M＋H]^＋^: 433.0664, found 433.0666.


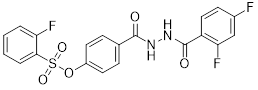
**4-(2-(2,6-difluorobenzoyl)hydrazine-1-carbonyl)phenyl-2-fluorobenzenesulfonate(A_2_)**: White solid, m.p.212-214 ℃; Yield: 75%. ^1^H NMR (400 MHz, DMSO) *δ* 10.78 (d, *J* = 13.2 Hz, 2H, -NH-NH-), 8.01 – 7.88 (m, 3H, Ph-H), 7.82 (td, *J* = 7.9, 1.7 Hz, 1H, Ph-H), 7.69 – 7.54 (m, 2H, Ph-H), 7.48 – 7.41 (m, 1H, Ph-H), 7.33 – 7.17 (m, 4H, Ph-H). ^13^C NMR (100 MHz, DMSO) *δ* 164.10, 160.39 (d, *J* = 7.6 Hz), 159.92, 159.15, 157.91 (d, *J* = 7.6 Hz), 157.35, 151.10, 138.58 (d, *J* = 8.9 Hz), 132.59 (t, *J* = 9.9 Hz), 131.63, 131.26, 129.84, 125.75 (d, *J* = 3.6 Hz), 122.09, 118.21, 118.01, 113.30 (t, *J* = 22.5 Hz), 112.25, 112.01. HRMS (ESI) calcd for C_20_H_14_O_5_N_2_F_3_S [M＋H]^＋^: 451.0570, found 451.0573.


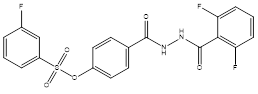
**4-(2-(2,6-difluorobenzoyl)hydrazine-1-carbonyl)phenyl-3-fluorobenzenesulfonate(A_3_)**: White solid, m.p.249-251 ℃; Yield: 73%. ^1^H NMR (400 MHz, DMSO) *δ* 10.78 (d, *J* = 11.0 Hz, 2H, -NH-NH-), 8.01 – 7.90 (m, 2H, Ph-H), 7.87 – 7.81 (m, 1H, Ph-H), 7.77 – 7.70 (m, 3H, Ph-H), 7.63 – 7.54 (m, 1H, Ph-H), 7.30 – 7.18 (m, 4H, Ph-H). ^13^C NMR (100 MHz, DMSO) *δ* 164.12, 163.13, 160.64, 160.39 (d, *J* = 7.7 Hz), 159.17, 157.91 (d, *J* = 7.6 Hz), 151.23, 135.77 (d, *J* = 7.5 Hz), 132.59, 132.42 (d, *J* = 8.2 Hz), 131.50, 129.75, 124.86 (d, *J* = 3.2 Hz), 122.74 (d, *J* = 21.2 Hz), 122.36, 115.51 (d, *J* = 25.2 Hz), 113.31 (t, *J* = 22.6 Hz), 112.25, 112.01. HRMS (ESI) calcd for C_20_H_14_O_5_N_2_F_3_S [M＋H]^＋^: 451.0570, found 451.0573.


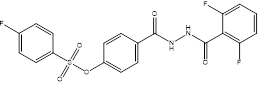
**4-(2-(2,6-difluorobenzoyl)hydrazine-1-carbonyl)phenyl-4-fluorobenzenesulfonate(A_4_)**: White solid, m.p.224-226 ℃; Yield: 78%. ^1^H NMR (400 MHz, DMSO) *δ* 10.78 (d, *J* = 10.4 Hz, 2H, -NH-NH-), 8.00 – 7.91 (m, 4H, Ph-H), 7.62 – 7.50 (m, 3H, Ph-H), 7.30 – 7.18 (m, 4H, Ph-H). ^13^C NMR (100 MHz, DMSO) *δ* 167.04, 164.51, 164.12, 160.40 (d, *J* = 7.7 Hz), 159.19, 157.92 (d, *J* = 7.6 Hz), 151.29, 132.60 (t, *J* = 9.9 Hz), 131.81 (d, *J* = 10.2 Hz), 131.43 130.24 (d, *J* = 3.0 Hz), 129.72, 122.44, 117.53 117.30, 113.32 (t, *J* = 22.5 Hz), 112.26, 112.02. HRMS (ESI) calcd for C_20_H_14_O_5_N_2_F_3_S [M＋H]^＋^: 451.0570, found 451.0574.


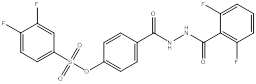
**4-(2-(2,6-difluorobenzoyl)hydrazine-1-carbonyl)phenyl-3,4-difluorobenzenesulfonate(A_5_)**: White solid, m.p.195-197 ℃; Yield: 66%. ^1^H NMR (400 MHz, DMSO) *δ* 10.79 (d, *J* = 12.5 Hz, 2H, -NH-NH-), 8.25 – 8.12 (m, 1H, Ph-H), 8.01 – 7.91 (m, 2H, Ph-H), 7.79 – 7.72 (m, 2H, Ph-H), 7.64 – 7.53 (m, 1H, Ph-H), 7.31 – 7.16 (m, 4H, Ph-H). ^13^C NMR (100 MHz, DMSO) *δ* 164.11, 160.40 (d, *J* = 7.6 Hz), 159.18, 157.92 (d, *J* = 7.7 Hz), 155.06 (d, *J* = 12.4 Hz), 152.51 (d, *J* = 12.3 Hz), 151.18, 148.49 (d, *J* = 13.6 Hz), 132.59 (t, *J* = 9.9 Hz), 131.56, 130.80, 129.79, 126.90 (d, *J* = 4.8 Hz), 122.49, 119.42 (d, *J* = 18.8 Hz), 118.71, 118.50, 113.32 (t, *J* = 22.5 Hz), 112.25, 112.01. HRMS (ESI) calcd for C_20_H_13_O_5_N_2_F_4_S [M＋H]^＋^: 469.0476, found 469.0478.


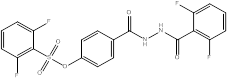
**4-(2-(2,6-difluorobenzoyl)hydrazine-1-carbonyl)phenyl-2,6-difluorobenzenesulfonate(A_6_)**: White solid, m.p.238-240 ℃; Yield: 68%. ^1^H NMR (400 MHz, DMSO) *δ* 10.80 (d, *J* = 19.8 Hz, 2H, -NH-NH-), 8.03 – 7.88 (m, 3H, Ph-H), 7.64 – 7.54 (m, 1H, Ph-H), 7.45 (t, *J* = 8.8 Hz, 2H, Ph-H), 7.37 – 7.28 (m, 2H, Ph-H), 7.23 (t, *J* = 8.1 Hz, 2H, Ph-H). ^13^C NMR (100 MHz, DMSO) *δ* 164.11, 160.44, 160.39 (d, *J* = 3.2 Hz), 159.17, 157.92 (d, *J* = 7.7 Hz), 157.80 (d, *J* = 3.0 Hz), 150.94, 138.98 (t, *J* = 11.4 Hz), 132.60 (t, *J* = 10.0 Hz), 131.87, 129.97, 121.97, 114.30 (d, *J* = 2.2 Hz), 114.07 (d, *J* = 1.9 Hz), 113.31 (t, *J* = 22.6 Hz), 112.26, 112.02, 111.81 (t, *J* = 15.5 Hz). HRMS (ESI) calcd for C_20_H_13_O_5_N_2_F_4_S [M＋H]^＋^: 469.04758, found 469.04831.


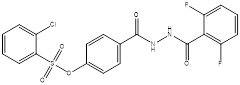
**4-(2-(2,6-difluorobenzoyl)hydrazine-1-carbonyl)phenyl-2-chlorobenzenesulfonate(A_7_)**: White solid, m.p.209-211 ℃; Yield:71%. ^1^H NMR (500 MHz, DMSO) *δ* 10.77 (d, *J* = 11.8 Hz, 2H, -NH-NH-), 7.94 (ddd, *J* = 4.9, 3.6, 1.9 Hz, 3H, Ph-H), 7.90 (dd, *J* = 8.1, 1.2 Hz, 1H, Ph-H), 7.86 – 7.80 (m, 2H, Ph-H), 7.61 – 7.54 (m, 2H, Ph-H), 7.30 – 7.25 (m, 2H, Ph-H), 7.25 – 7.19 (m, 2H, Ph-H). ^13^C NMR (125 MHz, DMSO) *δ* 164.12, 160.14 (d, *J* = 7.5 Hz), 159.16, 158.16 (d, *J* = 7.6 Hz), 151.12, 136.90, 132.60 (d, *J* = 33.0 Hz), 131.92, 131.60, 129.87, 128.40, 122.03, 113.30 (t, *J* = 22.6 Hz), 112.23, 112.04. HRMS (ESI) calcd for C_20_H_14_O_5_N_2_ClF_2_S [M＋H]^＋^: 467.0275, found 467.0278.


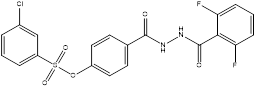
**4-(2-(2,6-difluorobenzoyl)hydrazine-1-carbonyl)phenyl-3-chlorobenzenesulfonate(A_8_)**: White solid, m.p.206-208 ℃; Yield: 75%. ^1^H NMR (500 MHz, DMSO) *δ* 10.79 (d, *J* = 16.0 Hz, 2H, -NH-NH-), 8.03 – 7.91 (m, 4H, Ph-H), 7.85 (ddd, *J* = 7.9, 1.7, 1.0 Hz, 1H, Ph-H), 7.72 (t, *J* = 8.0 Hz, 1H, Ph-H), 7.63 – 7.55 (m, 1H, Ph-H), 7.25 (ddd, *J* = 18.4, 11.5, 5.1 Hz, 4H, Ph-H). ^13^C NMR (125 MHz, DMSO) *δ* 164.11, 160.15 (d, *J* = 7.6 Hz), 159.17, 158.16 (d, *J* = 7.5 Hz), 151.19, 135.76, 135.42, 134.64, 132.60 (t, *J* = 9.8 Hz), 131.98, 131.53, 129.77, 127.74, 127.23, 122.40, 113.32 (t, *J* = 22.5 Hz), 112.24, 112.04. HRMS (ESI) calcd for C_20_H_14_O_5_N_2_ClF_2_S [M＋H]^＋^: 467.0275, found 467.0278.


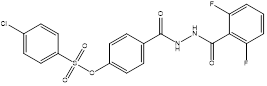
**4-(2-(2,6-difluorobenzoyl)hydrazine-1-carbonyl)phenyl-4-chlorobenzenesulfonate(A_9_)**: White solid, m.p.212-214 ℃; Yield:79%. ^1^H NMR (500 MHz, DMSO) *δ* 10.78 (d, *J* = 13.5 Hz, 2H, -NH-NH-), 7.99 – 7.93 (m, 2H, Ph-H), 7.92 – 7.87 (m, 2H, Ph-H), 7.79 – 7.74 (m, 2H, Ph-H), 7.63 – 7.55 (m, 1H, Ph-H), 7.30 – 7.15 (m, 4H, Ph-H). ^13^C NMR (125 MHz, DMSO) *δ* 164.11, 160.15 (d, *J* = 7.5 Hz), 159.19, 158.16 (d, *J* = 7.6 Hz), 151.24, 140.42, 132.76, 132.60 (t, *J* = 10.0 Hz), 131.48, 130.28 (d, *J* = 14.7 Hz), 129.76, 122.45, 113.32 (t, *J* = 22.6 Hz), 112.24, 112.04. C_20_H_12_O_5_N_2_ClF_2_S [M-H]^-^: 465.0118, found 465.0132.


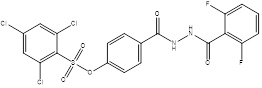
**4-(2-(2,6-difluorobenzoyl)hydrazine-1-carbonyl)phenyl-2,4,6-trichlorobenzenesulfonate(A_10_)**: White solid, m.p.239-241 ℃; Yield: 80%. ^1^H NMR (500 MHz, DMSO) *δ* 10.83 (s, 1H, -NH-), 10.77 (s, 1H, -NH-), 8.06 (s, 2H, Ph-H), 8.01 – 7.93 (m, 2H, Ph-H), 7.62 – 7.55 (m, 1H, Ph-H), 7.37 – 7.30 (m, 2H, Ph-H), 7.25 – 7.20 (m, 2H, Ph-H). ^13^C NMR (125 MHz, DMSO) *δ* 164.11, 160.14 (d, *J* = 7.6 Hz), 159.16, 158.16 (d, *J* = 7.7 Hz), 150.88, 140.20, 136.20, 132.60 (t, *J* = 9.6 Hz), 132.01, 131.83, 130.02, 129.26, 122.00, 113.30 (t, *J* = 22.6 Hz), 112.23, 112.04. HRMS (ESI) calcd for C_20_H_12_O_5_N_2_Cl_3_F_2_S [M＋H]^＋^: 534.9495, found 534.9503.


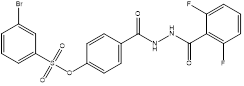
**4-(2-(2,6-difluorobenzoyl)hydrazine-1-carbonyl)phenyl-3-bromobenzenesulfonate(A_11_)**: White solid, m.p.194-196 ℃; Yield: 82%. ^1^H NMR (500 MHz, DMSO) *δ* 10.77 (d, *J* = 10.7 Hz, 2H, -NH-NH-), 8.07 (dd, *J* = 8.0, 1.1 Hz, 1H, Ph-H), 7.98 – 7.90 (m, 3H, Ph-H), 7.72 (td, *J* = 7.7, 1.7 Hz, 1H, Ph-H), 7.63 – 7.55 (m, 2H, Ph-H), 7.31 – 7.26 (m, 2H, Ph-H), 7.23 (t, *J* = 8.1 Hz, 2H, Ph-H). ^13^C NMR (125 MHz, DMSO) *δ* 164.07, 160.12 (d, *J* = 7.7 Hz), 159.12, 158.14 (d, *J* = 7.6 Hz), 151.15, 138.25, 135.87, 132.54 (t, *J* = 9.7 Hz), 132.10, 131.51, 130.37, 129.73, 127.49, 122.75, 122.34, 113.30 (t, *J* = 22.6 Hz), 112.19, 111.99. HRMS (ESI) calcd for C_20_H_14_O_5_N_2_BrF_2_S [M＋H]^＋^: 510.9770, found 510.9775.


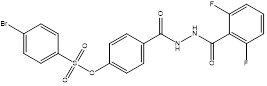
**4-(2-(2,6-difluorobenzoyl)hydrazine-1-carbonyl)phenyl-4-bromobenzenesulfonate(A_12_)**: White solid, m.p.206-208 ℃; Yield: 84%. ^1^H NMR (500 MHz, DMSO) *δ* 10.79 (d, *J* = 13.3 Hz, 2H, -NH-NH-), 7.98 – 7.88 (m, 4H, Ph-H), 7.83 – 7.78 (m, 2H, Ph-H), 7.59 (ddd, *J* = 14.8, 8.4, 6.5 Hz, 1H, Ph-H), 7.23 (dt, *J* = 4.6, 3.7 Hz, 4H, Ph-H). ^13^C NMR (125 MHz, DMSO) *δ* 164.11, 160.15 (d, *J* = 7.5 Hz), 159.19, 158.16 (d, *J* = 7.6 Hz), 151.24, 133.17, 132.61 (t, *J* = 9.7 Hz), 131.49, 130.30, 129.76, 129.65, 122.45, 113.32 (t, *J* = 22.6 Hz), 112.24, 112.05. HRMS (ESI) calcd for C_20_H_14_O_5_N_2_BrF_2_S_2_ [M＋H]^＋^: 510.9769, found 510.9774.


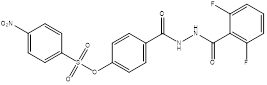
**4-(2-(2,6-difluorobenzoyl)hydrazine-1-carbonyl)phenyl-4-nitrobenzenesulfonate(A_13_)**: White solid, m.p.240-242 ℃; Yield: 71%. ^1^H NMR (500 MHz, DMSO) *δ* 10.79 (d, *J* = 17.5 Hz, 2H, -NH-NH-), 8.52 – 8.44 (m, 2H, Ph-H), 8.24 – 8.16 (m, 2H, Ph-H), 8.01 – 7.91 (m, 2H, Ph-H), 7.63 – 7.55 (m, 1H, Ph-H), 7.31 – 7.17 (m, 4H, Ph-H). ^13^C NMR (125 MHz, DMSO) *δ* 164.05, 160.15 (d, *J* = 7.6 Hz), 159.18, 158.16 (d, *J* = 7.4 Hz), 151.24, 151.08, 139.16, 132.61 (t, *J* = 9.7 Hz), 131.68, 130.20, 129.86, 125.22, 122.45, 113.30 (t, *J* = 22.5 Hz), 112.24, 112.04. HRMS (ESI) calcd for C_20_H_14_O_7_N_3_F_2_S [M＋H]^＋^: 478.05150, found 478.05215.


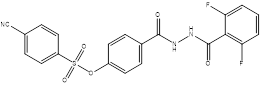
**4-(2-(2,6-difluorobenzoyl)hydrazine-1-carbonyl)phenyl-4-cyanobenzenesulfonate(A_14_)**: White solid, m.p.228-230 ℃; Yield: 66%. ^1^H NMR (500 MHz, DMSO) *δ* 10.79 (d, *J* = 15.3 Hz, 2H, -NH-NH-), 8.21 – 8.16 (m, 2H, Ph-H), 8.13 – 8.06 (m, 2H, Ph-H), 7.98 – 7.92 (m, 2H, Ph-H), 7.59 (ddd, *J* = 14.7, 8.3, 6.5 Hz, 1H, Ph-H), 7.30 – 7.15 (m, 4H, Ph-H). ^13^C NMR (125 MHz, DMSO) *δ* 164.07, 160.15 (d, *J* = 7.5 Hz), 159.18, 158.16 (d, *J* = 7.6 Hz), 151.09, 137.93, 134.10, 132.61 (t, *J* = 9.7 Hz), 131.64, 129.83, 129.18, 122.44, 117.60, 117.36, 113.31 (t, *J* = 22.6 Hz), 112.24, 112.05. HRMS (ESI) calcd for C_21_H_14_O_5_N_3_F_2_S [M＋H]^＋^: 458.0617, found 458.0619.


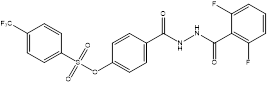
**4-(2-(2,6-difluorobenzoyl)hydrazine-1-carbonyl)phenyl-4-(trifluoromethyl)benzenesulfonate(A_15_)**: White solid, m.p.198-200 ℃; Yield: 65%. ^1^H NMR (500 MHz, DMSO) *δ* 10.74 (d, *J* = 16.2 Hz, 2H, -NH-NH-), 8.07 (dd, *J* = 24.4, 8.5 Hz, 4H, Ph-H), 7.92 (d, *J* = 8.8 Hz, 2H, Ph-H), 7.58 – 7.51 (m, 1H, Ph-H), 7.29 – 7.13 (m, 4H, Ph-H). ^13^C NMR (125 MHz, DMSO) *δ* 164.14, 160.15 (d, *J* = 7.7 Hz), 159.20, 158.60, 158.17 (d, *J* = 7.7 Hz), 151.43, 132.61 (t, *J* = 9.7 Hz), 131.25 (d, *J* = 8.2 Hz), 129.66, 128.28, 126.88, 122.31, 113.32 (t, *J* = 22.4 Hz), 112.25, 112.05, 35.28, 30.70. HRMS (ESI) calcd for C_21_H_14_O_5_N_2_F_5_S [M＋H]^＋^: 501.05381, found 501.05432.


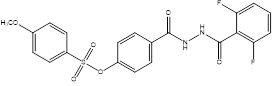
**4-(2-(2,6-difluorobenzoyl)hydrazine-1-carbonyl)phenyl-4-methoxybenzenesulfonate(A_16_)**: White solid, m.p.184-186 ℃; Yield: 78%. ^1^H NMR (500 MHz, DMSO) *δ* 13.68 (s, 1H, -NH-), 8.02 (d, *J* = 8.8 Hz, 2H, Ph-H), 7.83 (d, *J* = 9.0 Hz, 2H, Ph-H), 7.74 – 7.63 (m, 1H, Ph-H), 7.31 (t, *J* = 8.2 Hz, 2H, Ph-H), 7.23 – 7.15 (m, 4H, Ph-H), 3.87 (s, 3H, -OCH3). ^13^C NMR (125 MHz, DMSO) *δ* 164.24, 161.39, 160.09 (d, *J* = 6.7 Hz), 158.86, 158.27, 158.09 (d, *J* = 6.7 Hz), 150.56, 133.86, 130.84, 129.05, 128.94, 125.21, 123.30, 115.16, 112.52, 112.33, 56.04. HRMS (ESI) calcd for C_21_H_17_O_6_N_2_F_2_S [M＋H]^＋^: 463.0770, found 463.0773.


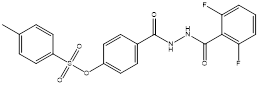
**4-(2-(2,6-difluorobenzoyl)hydrazine-1-carbonyl)phenyl-4-methylbenzenesulfonate(A_17_)**: White solid, m.p.204-206 ℃; Yield: 69%. ^1^H NMR (500 MHz, DMSO) *δ* 10.77 (d, *J* = 7.9 Hz, 2H, -NH-NH-), 7.92 (d, *J* = 8.8 Hz, 2H, Ph-H), 7.76 (t, *J* = 5.2 Hz, 2H, Ph-H), 7.65 – 7.53 (m, 2H, Ph-H), 7.49 (d, *J* = 8.1 Hz, 2H, Ph-H), 7.30 – 7.15 (m, 2H, Ph-H), 2.42 (s, 2H, Ph-H). ^13^C NMR (125 MHz, DMSO) *δ* 164.15, 160.15 (d, *J* = 7.7 Hz), 159.19, 158.16 (d, *J* = 7.6 Hz), 151.46, 146.19, 132.61, 131.23, 131.06, 130.44, 129.65, 128.41, 122.35, 113.32 (t, *J* = 22.5 Hz), 112.25, 112.06, 21.28. HRMS (ESI) calcd for C_21_H_17_O_5_N_2_F_2_S [M＋H]^＋^: 447.08208, found 447.08224.


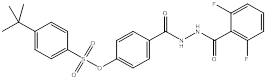
**4-(2-(2,6-difluorobenzoyl)hydrazine-1-carbonyl)phenyl-4-(tert-butyl)benzenesulfonate(A_18_)**: White solid, m.p.178-180 ℃; Yield: 80%. ^1^H NMR (500 MHz, DMSO) *δ* 10.78 (d, *J* = 10.4 Hz, 2H, -NH-NH-), 7.94 (d, *J* = 8.8 Hz, 2H, Ph-H), 7.82 (d, *J* = 8.6 Hz, 2H, Ph-H), 7.71 (d, *J* = 8.7 Hz, 2H, Ph-H), 7.59 (ddd, *J* = 15.2, 8.4, 6.7 Hz, 1H, Ph-H), 7.22 (dd, *J* = 15.3, 8.4 Hz, 4H, Ph-H), 1.31 (s, 9H,-C(CH3)3). ^13^C NMR (125 MHz, DMSO) *δ* 164.14, 160.15 (d, *J* = 7.7 Hz), 159.20, 158.60, 158.17 (d, *J* = 7.7 Hz), 151.43, 132.61 (t, *J* = 9.7 Hz), 131.25 (d, *J* = 8.2 Hz), 129.66, 128.28, 126.88, 122.31, 113.32 (t, *J* = 22.4 Hz), 112.25, 112.05, 35.28, 30.70. HRMS (ESI) calcd for C_24_H_23_O_5_N_2_F_2_S [M＋H]^＋^: 489.1290, found 489.1296.


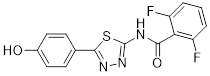
**2,6-difluoro-N-(5-(4-hydroxyphenyl)-1,3,4-thiadiazol-2-yl)benzamide(中间体B-3)**: Yellow solid, m.p. 185-187 ℃; Yield: 61.4%. ^1^H NMR (400 MHz, DMSO) *δ* 13.50 (s, 1H, -NH-), 10.17 (s, 1H, -OH), 7.81 (d, *J* = 8.7 Hz, 2H, Ph-H), 7.74 – 7.59 (m, 1H, Ph-H), 7.30 (t, *J* = 8.2 Hz, 2H, Ph-H), 6.91 (d, *J* = 8.7 Hz, 2H, Ph-H). ^13^C NMR (100 MHz, DMSO) *δ* 160.33 (d, *J* = 6.9 Hz), 160.01, 157.84 (d, *J* = 6.9 Hz), 133.69 (t, *J* = 10.3 Hz), 128.92, 120.94, 116.20, 112.49, 112.30. HRMS (ESI) calcd for C_15_H_10_O_2_N_3_F_2_S [M＋H]^＋^: 334.0456, found 334.0455.


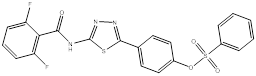
**4-(5-(2,6-difluorobenzamido)-1,3,4-thiadiazol-2-yl)phenyl-benzenesulfonate(B_1_):** White solid, m.p.265-267 ℃; Yield: 72%. ^1^H NMR (500 MHz, DMSO) *δ* 13.64 (s, -NH-), 8.07 – 7.99 (m, 2H, Ph-H), 7.93 (dt, *J* = 8.6, 1.5 Hz, 2H, Ph-H), 7.88 – 7.81 (m, 1H, Ph-H), 7.74 – 7.63 (m, 3H, Ph-H), 7.30 (t, *J* = 8.3 Hz, 2H, Ph-H), 7.25 – 7.18 (m, 2H, Ph-H ).^13^C NMR (125 MHz, DMSO) *δ* 161.28, 160.04 (d, *J* = 6.7 Hz), 158.80, 158.22, 158.05 (d, J = 6.8 Hz), 150.38, 135.30, 134.10, 133.78 (t, J = 10.1 Hz), 129.98, 129.15, 128.92 , 128.30, 123.16, 112.44, 112.25. HRMS (ESI) calcd for C_21_H_14_O_4_N_3_F_2_S_2_ [M＋H]^＋^: 474.0388, found 474.0385.


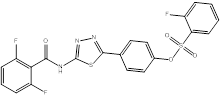
**4-(5-(2,6-difluorobenzamido)-1,3,4-thiadiazol-2-yl)phenyl-2-fluorobenzenesulfonate(B_2_):** White solid, m.p.266-268 ℃; Yield: 74%. ^1^H NMR (500 MHz, DMSO) *δ* 13.65 (s, 1H, -NH-), 8.10 – 8.00 (m, 2H, Ph-H), 7.98 – 7.88 (m, 1H, Ph-H), 7.88 – 7.81 (m, 1H, Ph-H), 7.74 – 7.62 (m, 2H, Ph-H), 7.46 (td, *J* = 7.8, 1.0 Hz, 1H, Ph-H), 7.38 – 7.25 (m, 4H, Ph-H). ^13^C NMR (125 MHz, DMSO) *δ* 161.19 , 160.04 (d, *J* = 6.6 Hz), 159.62, 158.83, 158.28, 158.05 (d, *J* = 6.8 Hz), 157.57 , 150.11, 138.52 (d, *J* = 8.7 Hz), 133.78 (t, *J* = 10.2 Hz), 131.22, 129.43, 129.09, 125.73 (d, *J* = 3.2 Hz), 122.89, 122.03 (d, *J* = 13.4 Hz), 118.06 (d, *J* = 20.3 Hz), 112.45, 112.25.. HRMS (ESI) calcd for C_21_H_14_O_4_N_3_F_2_S_2_ [M＋H]^＋^: 492.0294, found 492.0289.


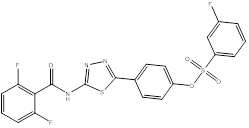
**4-(5-(2,6-difluorobenzamido)-1,3,4-thiadiazol-2-yl)phenyl-3-fluorobenzenesulfonate(B_3_):** White solid, m.p.240-242 ℃; Yield: 70%. ^1^H NMR (500 MHz,DMSO ) *δ* 13.65 (s, 1H, -NH-), 8.08 – 8.01 (m, 2H, Ph-H), 7.88 – 7.83 (m, 1H, Ph-H), 7.78 – 7.73 (m, 3H, Ph-H), 7.72 – 7.63 (m, 1H, Ph-H), 7.35 – 7.24 (m, 4H, Ph-H). ^13^C NMR (125 MHz, DMSO) *δ* 161.19, 160.04 (d, *J* = 6.6 Hz), 159.62, 158.83, 158.28, 158.05 (d, *J* = 6.8 Hz), 157.57, 150.11, 138.52 (d, *J* = 8.7 Hz), 133.78 (t, *J* = 10.2 Hz), 131.22, 129.43, 129.09, 125.73 (d, *J* = 3.2 Hz), 122.89, 122.03 (d, *J* = 13.4 Hz), 118.06 (d, *J* = 20.3 Hz), 112.45, 112.25. HRMS (ESI) calcd for C_21_H_14_O_4_N_3_F_2_S_2_ [M＋H]^＋^: 492.0294, found 492.0290.


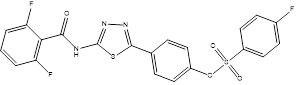
**4-(5-(2,6-difluorobenzamido)-1,3,4-thiadiazol-2-yl)phenyl-4-fluorobenzenesulfonate(B_4_):** White solid, m.p.261-263 ℃; Yield: 79%. ^1^H NMR (500 MHz, DMSO) *δ* 13.65 (s, 1H, -NH-), 8.06 – 7.97 (m, 4H, Ph-H), 7.74 – 7.63 (m, 1H, Ph-H), 7.58 – 7.51 (m, 2H, Ph-H), 7.36 – 7.21 (m, 4H, Ph-H). ^13^C NMR (125 MHz, DMSO) *δ* 166.75, 164.72, 161.27, 160.04 (d, *J* = 6.8 Hz), 158.80, 158.26, 158.05 (d, *J* = 6.7 Hz), 150.29, 133.78 (t, *J* = 10.5 Hz), 131.80, 131.72, 130.34 (d, *J* = 2.6 Hz), 129.25, 128.97, 123.25, 117.47, 117.28, 112.45, 112.26. HRMS (ESI) calcd for C_21_H_14_O_4_N_3_F_2_S_2_ [M＋H]^＋^: 492.0294, found 492.0291.


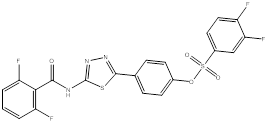
**4-(5-(2,6-difluorobenzamido)-1,3,4-thiadiazol-2-yl)phenyl-3,4-difluorobenzenesulfonate(B_5_):** White solid, m.p.244-246 ℃; Yield: 69%. ^1^H NMR (500 MHz, DMSO) *δ* 13.65 (s, 1H, -NH-), 8.24 – 8.13 (m, 1H, Ph-H), 8.07 – 7.96 (m, 2H, Ph-H), 7.85 – 7.63 (m, 3H, Ph-H), 7.40 – 7.18 (m, 4H, Ph-H). ^13^C NMR (125 MHz, DMSO) *δ* 168.80, 161.25, 160.05 (d, *J* = 6.7 Hz), 158.83, 158.27, 158.06 (d, *J* = 6.8 Hz), 154.76 (d, *J* = 12.6 Hz), 152.72 (d, *J* = 12.4 Hz), 150.70 (d, *J* = 13.4 Hz), 150.17, 148.69 (d, *J* = 13.4 Hz), 133.79 (t, *J* = 9.8 Hz), 130.90, 129.39, 129.03, 128.75, 126.86 (d, *J* = 5.0 Hz), 123.30, 119.40 (d, *J* = 18.8 Hz), 118.64, 118.48, 112.45, 112.26. HRMS (ESI) calcd for C_21_H_14_O_4_N_3_F_2_S_2_ [M＋H]^＋^: 510.0200, found 510.0198.


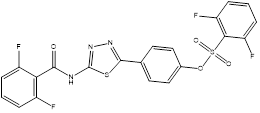
**4-(5-(2,6-difluorobenzamido)-1,3,4-thiadiazol-2-yl)phenyl-2,6-difluorobenzenesulfonate(B_6_):** White solid, m.p.273-275 ℃; Yield: 66%. ^1^H NMR (500 MHz, DMSO) *δ* 13.65 (s, 1H,-NH-), 8.18 – 7.99 (m, 2H, Ph-H), 7.99 – 7.90 (m, 1H, Ph-H), 7.75 – 7.63 (m, 1H, Ph-H), 7.46 (t, *J* = 8.8 Hz, 2H, Ph-H), 7.39 – 7.24 (m, 4H, Ph-H). **^13^**C NMR (125 MHz, DMSO) *δ* 161.65 , 160.58, 160.52, 159.33, 158.84, 158.58 , 158.53, 150.45, 139.42 (t, *J* = 11.3 Hz), 134.29 (t, *J* = 10.7 Hz), 130.15, 129.71, 123.24, 114.75 (d, *J* = 3.3 Hz), 114.58 (d, *J* = 3.8 Hz), 112.95, 112.76, 112.56, 112.44, 112.31. HRMS (ESI) calcd for C_21_H_14_O_4_N_3_F_2_S_2_ [M＋H]^＋^: 510.0200, found 510.0198.


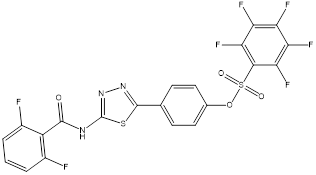
**4-(5-(2,6-difluorobenzamido)-1,3,4-thiadiazol-2-yl)phenyl-pentafluorobenzenesulfonate(B_7_):** White solid, m.p.241-243℃; Yield: 75%. ^1^H NMR (400 MHz, DMSO) *δ* 13.71 (s, 1H), 8.18 – 8.01 (m, 2H), 7.75 – 7.61 (m, 1H), 7.58 – 7.53 (m, 1H), 7.51 – 7.39 (m, 1H), 7.31 (td, *J* = 8.3, 2.5 Hz, 1H), 7.25 – 7.12 (m, 1H). ^13^C NMR (100 MHz, DMSO) *δ* 161.25, 160.05 (d, *J* = 6.7 Hz), 158.83, 158.06 (d, *J* = 6.8 Hz), 154.76 (d, *J* = 12.6 Hz), 152.72 (d, *J* = 12.4 Hz), 150.70 (d, *J* = 13.4 Hz), 150.17, 148.69 (d, *J* = 13.4 Hz), 133.79, 130.90, 129.39, 129.03, 128.75, 126.86 (d, *J* = 5.0 Hz), 123.30, 119.40 (d, *J* = 18.8 Hz), 118.64, 118.48, 112.45, 112.26. HRMS (ESI) calcd for C_21_H_14_O_4_N_3_F_2_S_2_ [M＋H]^＋^: 563.9917, found 563.9916.


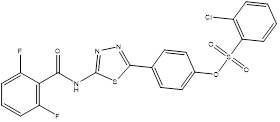
**4-(5-(2,6-difluorobenzamido)-1,3,4-thiadiazol-2-yl)phenyl-2-chlorobenzenesulfonate(B_8_):** White solid, m.p.275-277 ℃; Yield: 81%. ^1^H NMR (500 MHz, DMSO) *δ* 13.64 (s, 1H, -NH-), 8.06 – 8.01 (m, 2H, Ph-H), 7.98 (dd, *J* = 8.0, 1.6 Hz, 1H, Ph-H), 7.93 – 7.80 (m, 2H, Ph-H), 7.74 – 7.63 (m, 1H, Ph-H), 7.63 – 7.55 (m, 1H, Ph-H), 7.38 – 7.24 (m, 4H, Ph-H). ^13^C NMR (125 MHz, DMSO) *δ* 161.18, 160.04 (d, *J* = 6.6 Hz), 158.82, 158.27, 158.04 (d, *J* = 6.8 Hz), 150.13, 136.84, 133.78 (t, *J* = 9.5 Hz), 132.68, 132.42, 132.00, 131.85, 129.37, 129.12, 128.38, 122.83, 112.44, 112.25. HRMS (ESI) calcd for C_21_H_14_O_4_N_3_F_2_S_2_ [M＋H]^＋^: 507.9999, found 507.9999.


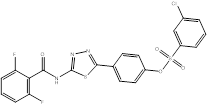
**4-(5-(2,6-difluorobenzamido)-1,3,4-thiadiazol-2-yl)phenyl-3-chlorobenzenesulfonate(B_9_):** White solid, m.p.227-229 ℃; Yield: 78%. ^1^H NMR (500 MHz, DMSO) *δ* 13.65 (s, 1H, -NH-), 8.09 – 7.85 (m, 5H, Ph-H), 7.77 – 7.61 (m, 2H, Ph-H), 7.38 – 7.19 (m, 4H, Ph-H). ^13^C NMR (125 MHz, DMSO) *δ* 161.24, 160.29 (d, *J* = 6.8 Hz), 158.82, 158.28, 157.79 (d, *J* = 6.7 Hz), 150.18, 135.86, 135.35, 134.59, 133.77, 131.94, 129.35, 129.01, 127.68, 127.16, 123.19, 112.46, 112.22. HRMS (ESI) calcd for C_21_H_14_O_4_N_3_F_2_S_2_ [M＋H]^＋^: 507.9999, found 507.9998.


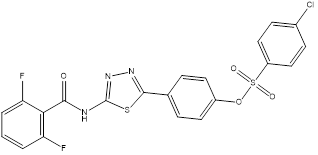
**4-(5-(2,6-difluorobenzamido)-1,3,4-thiadiazol-2-yl)phenyl-4-chlorobenzenesulfonate(B_10_):** White solid, m.p.257-259 ℃; Yield: 79%. ^1^H NMR (500 MHz, DMSO) *δ* 13.65 (s, 1H, -NH-), 8.14 – 8.00 (m, 2H, Ph-H), 8.00 – 7.89 (m, 2H, Ph-H), 7.81 – 7.73 (m, 2H, Ph-H), 7.73 – 7.63 (m, 1H, Ph-H), 7.36 – 7.22 (m, 4H, Ph-H). ^13^C NMR (125 MHz, DMSO) *δ* 161.25, 160.29 (d, *J* = 6.8 Hz), 158.84, 158.27, 157.80 (d, *J* = 6.8 Hz), 150.24, 140.36, 133.77, 132.87, 130.27, 130.18, 129.30, 129.01, 123.23, 112.47, 112.22. HRMS (ESI) calcd for C_21_H_14_O_4_N_3_F_2_S_2_ [M＋H]^＋^: 507.9999, found 507.9999.


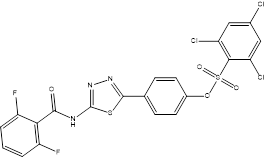
**4-(5-(2,6-difluorobenzamido)-1,3,4-thiadiazol-2-yl)phenyl-2,4,6-trichlorobenzenesulfonate(B_11_):** White solid, m.p.269-271 ℃; Yield: 82%. ^1^H NMR (400 MHz, DMSO) *δ* 13.65 (s, 1H, -NH-), 8.13 – 8.00 (m, 4H, Ph-H), 7.73 – 7.62 (m, 1H, Ph-H), 7.45 – 7.20 (m, 4H, Ph-H). ^13^C NMR (100 MHz, DMSO) *δ* 160.29 (d, *J* = 6.9 Hz), 157.79 (d, *J* = 6.7 Hz), 149.89, 140.15, 136.14, 131.95, 129.58, 129.34, 129.24, 122.72, 112.47, 112.22. HRMS (ESI) calcd for C_21_H_14_O_4_N_3_F_2_S_2_ [M＋H]^＋^: 575.9219, found 575.9219.


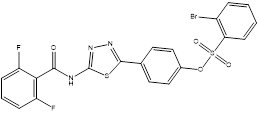
**4-(5-(2,6-difluorobenzamido)-1,3,4-thiadiazol-2-yl)phenyl-2-bromobenzenesulfonate(B_12_):** White solid, m.p.230-232 ℃; Yield: 78%. ^1^H NMR (400 MHz, DMSO) *δ* 13.64 (s, 1H, -NH-), 8.10 – 8.01 (m, 3H, Ph-H), 8.00 – 7.97 (m, 1H, Ph-H), 7.75 – 7.60 (m, 3H, Ph-H), 7.37 – 7.22 (m, 4H, Ph-H). ^13^C NMR (100 MHz, DMSO) *δ* 168.78, 161.18, 160.28 (d, *J* = 6.8 Hz), 158.81, 158.27, 157.79 (d, *J* = 6.9 Hz), 150.15, 136.64, 136.19, 133.86, 132.66, 129.33, 129.10, 128.77, 122.85, 120.43, 112.46, 112.22. HRMS (ESI) calcd for C_21_H_14_O_4_N_3_F_2_S_2_ [M＋H]^＋^: 551.9493, found 551.9493.


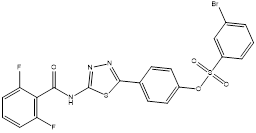
**4-(5-(2,6-difluorobenzamido)-1,3,4-thiadiazol-2-yl)phenyl-3-bromobenzenesulfonate(B_13_):** White solid, m.p.223-225 ℃; Yield: 79%. ^1^H NMR (400 MHz, DMSO) *δ* 13.65 (s, 1H, -NH-), 8.11 – 7.96 (m, 4H, Ph-H), 7.96 – 7.82 (m, 1H, Ph-H), 7.75 – 7.62 (m, 2H, Ph-H), 7.39 – 7.25 (m, 4H, Ph-H). ^13^C NMR (100 MHz, DMSO) *δ* 161.23, 160.29 (d, *J* = 6.8 Hz), 158.82, 158.26, 157.79 (d, *J* = 6.9 Hz), 150.17, 138.23, 135.96, 133.78, 132.10, 130.36, 129.35, 129.01, 127.47, 123.20, 122.74, 112.46 , 112.22. HRMS (ESI) calcd for C_21_H_14_O_4_N_3_F_2_S_2_ [M＋H]^＋^: 551.9493, found 551.9494.


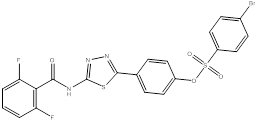
**4-(5-(2,6-difluorobenzamido)-1,3,4-thiadiazol-2-yl)phenyl-4-bromobenzenesulfonate(B_14_):** White solid, m.p.250-252 ℃; Yield: 76%. ^1^H NMR (400 MHz, DMSO) *δ* 13.65 (s, 1H, -NH-), 8.14 – 8.00 (m, 2H, -Ph-H), 7.98 – 7.88 (m2H, -Ph-H), 7.88 – 7.74 (m, 2H, -Ph-H), 7.73 – 7.63 (m, 1H, -Ph-H), 7.37 – 7.16 (m, 4H, -Ph-H). ^13^C NMR (100 MHz, DMSO) *δ* 161.25, 160.29 (d, *J* = 6.8 Hz), 158.82, 158.26, 157.80 (d, *J* = 6.8 Hz), 150.24, 133.77, 133.30, 133.12, 130.23, 129.57, 129.30, 129.01, 123.22, 112.47, 112.23. HRMS (ESI) calcd for C_21_H_14_O_4_N_3_F_2_S_2_ [M＋H]^＋^: 551.9493, found 551.9493.


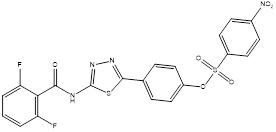
**4-(5-(2,6-difluorobenzamido)-1,3,4-thiadiazol-2-yl)phenyl-4-nitrobenzenesulfonate(B_15_):** White solid, m.p.278-280 ℃; Yield: 72%, ^1^H NMR (400 MHz, DMSO) *δ* 13.65 (s, 1H, -NH-), 8.53 – 8.43 (m, 2H, -Ph-H), 8.27 – 8.10 (m, 2H, -Ph-H), 8.10 – 8.00 (m, 2H, -Ph-H), 7.74 – 7.63 (m, 1H, -Ph-H), 7.38 – 7.16 (m, 4H, -Ph-H). ^13^C NMR (100 MHz, DMSO) *δ* 161.17, 160.29 (d, *J* = 6.8 Hz), 158.84, 158.29, 157.79 (d, *J* = 6.8 Hz), 151.19, 150.07, 139.26, 133.78, 130.16, 129.50, 129.09, 125.16, 123.23, 112.47, 112.23. HRMS (ESI) calcd for C_21_H_14_O_4_N_3_F_2_S_2_ [M＋H]^＋^: 519.0239, found 519.0239.


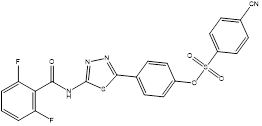
**4-(5-(2,6-difluorobenzamido)-1,3,4-thiadiazol-2-yl)phenyl-4-cyanobenzenesulfonate(B_16_):** White solid, m.p264.-266 ℃; Yield: 74%. ^1^H NMR (400 MHz, DMSO) *δ* 13.65 (s, 1H, -NH-), 8.20 – 8.16 (m, 2H, Ph-H), 8.14 – 8.09 (m, 2H, Ph-H), 8.06 – 7.96 (m, 2H, Ph-H), 7.74 – 7.62 (m, 1H, Ph-H), 7.38 – 7.20 (m, 4H, Ph-H). ^13^C NMR (100 MHz, DMSO) *δ* 168.80, 161.19, 160.29 (d, *J* = 6.7 Hz), 158.84, 158.29, 157.80 (d, *J* = 6.9 Hz), 150.09, 138.03, 134.06, 129.45 , 129.10 (d, *J* = 5.9 Hz), 128.79, 123.22, 117.55, 117.30, 112.47, 112.23. HRMS (ESI) calcd for C_21_H_14_O_4_N_3_F_2_S_2_ [M＋H]^＋^: 499.0341, found 499.0340.


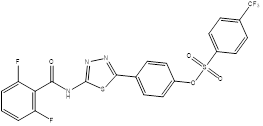
**4-(5-(2,6-difluorobenzamido)-1,3,4-thiadiazol-2-yl)phenyl-4-(trifluoromethyl)benzenesulfonate(B_17_):** White solid, m.p.248-250 ℃; Yield: 77%. ^1^H NMR (400 MHz, DMSO) *δ* 13.65 (s, 1H, -NH-), 8.16 (d, *J* = 8.3 Hz, 2H, Ph-H), 8.12 – 8.01 (m, 4H, Ph-H), 7.73 – 7.63 (m, 1H, Ph-H), 7.37 – 7.24 (m, 4H, Ph-H). ^13^C NMR (100 MHz, DMSO) *δ* 161.21, 160.29 (d, *J* = 6.7 Hz), 158.84, 158.31, 157.80 (d, *J* = 6.8 Hz), 150.14, 138.05, 134.66, 134.33, 133.78, 129.46, 129.07, 127.19 (d, *J* = 3.7 Hz), 124.51, 123.20, 121.80, 112.47, 112.23. HRMS (ESI) calcd for C_21_H_14_O_4_N_3_F_2_S_2_ [M＋H]^＋^: 542.0262, found 542.0261.


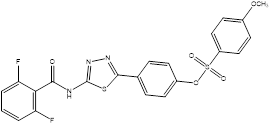
**4-(5-(2,6-difluorobenzamido)-1,3,4-thiadiazol-2-yl)phenyl-4-methoxybenzenesulfonate(B_18_):** White solid, m.p.245-247 ℃; Yield: 70%. ^1^H NMR (400 MHz, DMSO) *δ* 13.64 (s, 1H, -NH-), 8.05 – 7.98 (m, 2H, Ph-H), 7.86 – 7.80 (m, 2H, Ph-H), 7.73 – 7.63 (m, 1H, Ph-H), 7.30 (t, *J* = 8.2 Hz, 2H, Ph-H), 7.23 – 7.16 (m, 4H, Ph-H), 3.87 (s, 3H, -CH3). ^13^C NMR (100 MHz, DMSO) *δ* 164.20, 161.32, 160.29 (d, *J* = 6.8 Hz), 158.82, 158.22, 157.80 (d, *J* = 6.8 Hz), 150.52, 133.78, 130.76, 129.00, 128.88, 125.23, 123.22, 115.11, 112.47, 112.23, 55.99. HRMS (ESI) calcd for C_21_H_14_O_4_N_3_F_2_S_2_ [M＋H]^＋^: 504.04940, found 504.0491.


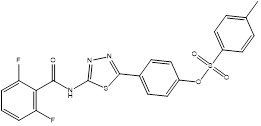
**4-(5-(2,6-difluorobenzamido)-1,3,4-thiadiazol-2-yl)phenyl-4-methylbenzenesulfonate(B1_9_):** White solid, m.p.220-222 ℃; Yield: 72%. ^1^H NMR (400 MHz, DMSO) *δ* 13.64 (s, 1H, -NH-), 8.11 – 7.93 (m, 2H,Ph-H), 7.84 – 7.74 (m, 2H,Ph-H), 7.73 – 7.62 (m, 1H,Ph-H), 7.49 (d, *J* = 8.0 Hz, 2H,Ph-H), 7.30 (t, *J* = 8.2 Hz, 2H,Ph-H), 7.25 – 7.15 (m, 2H,Ph-H), 2.42 (s, 3H,-CH3). ^13^C NMR (100 MHz, DMSO) *δ* 161.29, 160.30 (d, *J* = 6.8 Hz), 158.84, 158.25, 157.80 (d, *J* = 6.7 Hz), 150.46, 146.09, 133.76, 131.22, 130.38, 129.06, 128.90, 128.32, 123.15, 112.46, 112.22, 21.22. HRMS (ESI) calcd for C_21_H_14_O_4_N_3_F_2_S_2_ [M＋H]^＋^: 488.0545, found 488.0544.


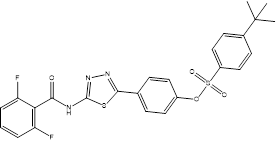
**4-(5-(2,6-difluorobenzamido)-1,3,4-thiadiazol-2-yl)phenyl-4-(tert-butyl)benzenesulfonate(B_20_):** White solid, m.p.272-274 ℃; Yield: 78%. ^1^H NMR (400 MHz, DMSO) *δ* 13.65 (s, 1H, -NH-), 8.17 – 7.95 (m, 2H, Ph-H), 7.95 – 7.82 (m, 1H, Ph-H), 7.77 – 7.63 (m, 3H, Ph-H), 7.38 – 7.14 (m, 4H, Ph-H), 1.30 (s, 9H, -C(CH3)3). ^13^C NMR (100 MHz, DMSO) *δ* 161.31 (s), 160.30 (d, *J* = 6.8 Hz), 158.83 (s), 158.52 (s), 158.40 – 157.50 (m), 150.44, 133.77, 131.42, 129.05, 128.91, 128.19, 126.81, 123.10, 112.46, 112.22, 35.19, 30.64. HRMS (ESI) calcd for C_21_H_14_O_4_N_3_F_2_S_2_ [M＋H]^＋^: 530.1014, found 530.1014.


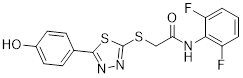
**N-(2,6-difluorophenyl)-2-((5-(4-hydroxyphenyl)-1,3,4-thiadiazol-2-yl)thio)acetamide(中间体C-4)**: Yellowish brown solid, m.p. 141-143 ℃; Yield: 67.1%. ^1^H NMR (500 MHz, DMSO) *δ* 10.27 (s, 1H, -NH-), 10.21 (s, 1H, -OH), 7.74 (d, *J* = 8.7 Hz, 2H, Ph-H), 7.43 – 7.30 (m, 1H, Ph-H), 7.17 (t, *J* = 8.2 Hz, 2H, Ph-H), 6.91 (d, *J* = 8.7 Hz, 2H, Ph-H), 4.37 (s, 2H, -CH2-). ^13^C NMR (125 MHz, DMSO) *δ* 168.50, 165.83, 162.84, 160.44, 158.63 (d, *J* = 5.1 Hz), 156.65 (d, *J* = 5.1 Hz), 129.36, 128.36 (t, *J* = 9.8 Hz), 120.31, 116.31, 114.09 (t, *J* = 16.9 Hz), 112.12, 111.94, 37.14. HRMS (ESI) calcd for C_16_H_12_O_2_N_3_F_2_S_2_ [M＋H]^＋^: 380.0334, found 380.0331.


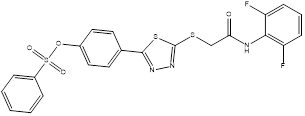
**4-(5-((2-((2,6-difluorophenyl)amino)-2-oxoethyl)thio)-1,3,4-thiadiazol-2-yl)phenyl-benzenesulfonate(C_1_)**: Yellow solid, m.p.140-142 ℃; Yield: 73%. ^1^H NMR (500 MHz, DMSO) *δ* 10.18 (s, 1H, -NH-), 7.97 – 7.89 (m, 4H, Ph-H), 7.87 – 7.81 (m, 1H, Ph-H), 7.72 – 7.66 (m, 2H, Ph-H), 7.40 – 7.32 (m, 1H, Ph-H), 7.26 – 7.20 (m, 2H, Ph-H), 7.16 (t, *J* = 8.2 Hz, 2H, Ph-H), 4.41 (s, 2H, -CH_2_-). ^13^C NMR (125 MHz, DMSO) *δ* 166.76, 165.64, 165.27, 158.58 (d, *J* = 5.0 Hz), 156.59 (d, *J* = 5.0 Hz), 150.68, 135.32, 134.02, 129.98, 129.31, 128.41, 128.29, 123.29, 114.01 (t, *J* = 16.7 Hz), 112.04, 111.85, 37.10. HRMS (ESI) calcd for C_21_H_14_O_4_N_3_F_2_S_2_ [M-H]^-^: 518.0109, found 518.0113.


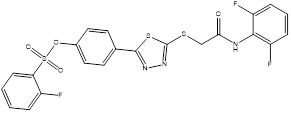
**4-(5-((2-((2,6-difluorophenyl)amino)-2-oxoethyl)thio)-1,3,4-thiadiazol-2-yl)phenyl-2-fluorobenzenesulfonate(C_2_)**: Yellow solid, m.p.118-120 ℃; Yield: 88%. ^1^H NMR (500 MHz, DMSO) *δ* 10.20 (s, 1H, -NH-), 8.09 – 7.86 (m, 3H, Ph-H), 7.83 (t, *J* = 7.0 Hz, 1H, Ph-H), 7.70 – 7.60 (m, 1H, Ph-H), 7.45 (t, *J* = 7.7 Hz, 1H, Ph-H), 7.37 (dd, *J* = 14.9, 7.8 Hz, 1H, Ph-H), 7.30 (d, *J* = 8.5 Hz, 2H, Ph-H), 7.16 (t, *J* = 8.2 Hz, 2H, Ph-H), 4.41 (s, 2H, -CH_2_-). ^13^C NMR (125 MHz, DMSO) *δ* 166.70, 165.68, 165.41, 159.64, 158.60 (d, *J* = 5.2 Hz), 157.59, 156.62 (d, *J* = 5.0 Hz), 150.42, 138.59 (d, *J* = 8.8 Hz), 131.25, 129.52, 128.72, 128.33 (t, *J* = 9.7 Hz), 125.76 (d, *J* = 3.3 Hz), 123.08, 121.96 (d, *J* = 13.5 Hz), 118.18, 118.02, 114.04 (t, *J* = 16.9 Hz), 112.08, 111.89, 37.12. HRMS (ESI) calcd for C_21_H_14_O_4_N_3_F_2_S_2_ [M-H]^-^: 536.0015, found 536.0021.


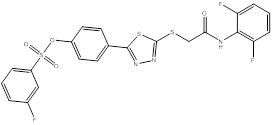
**4-(5-((2-((2,6-difluorophenyl)amino)-2-oxoethyl)thio)-1,3,4-thiadiazol-2-yl)phenyl-3-fluorobenzenesulfonate(C_3_)**: Yellow solid, m.p.133-135 ℃; Yield: 76%. ^1^H NMR (500 MHz, DMSO) *δ* 10.20 (s, 1H, -NH-), 8.01 – 7.91 (m, 2H, Ph-H), 7.87 – 7.82 (m, 1H, Ph-H), 7.77 – 7.71 (m, 3H, Ph-H), 7.36 (ddd, *J* = 14.7, 8.2, 6.3 Hz, 1H, Ph-H), 7.32 – 7.25 (m, 2H, Ph-H), 7.16 (t, *J* = 8.2 Hz, 2H, Ph-H), 4.41 (s, 2H, -CH_2_-). ^13^C NMR (125 MHz, DMSO) *δ* 166.76, 165.68, 165.38, 162.87, 160.89, 158.60 (d, *J* = 5.2 Hz), 156.62 (d, *J* = 5.1 Hz), 150.55, 135.79 (d, *J* = 7.5 Hz), 132.43 (d, *J* = 8.0 Hz), 129.43, 128.62, 128.34 (t, *J* = 9.7 Hz), 124.85 (d, *J* = 2.8 Hz), 123.35, 122.84, 122.67, 115.59, 115.39, 114.04 (t, *J* = 16.8 Hz), 112.08, 111.90, 37.12. HRMS (ESI) calcd for C_21_H_14_O_4_N_3_F_2_S_2_ [M-H]^-^: 536.0015, found 536.0023.


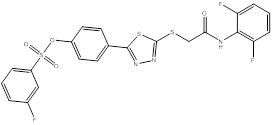
**4-(5-((2-((2,6-difluorophenyl)amino)-2-oxoethyl)thio)-1,3,4-thiadiazol-2-yl)phenyl-4-fluorobenzenesulfonate(C_4_)**: Yellow solid, m.p.140-142 ℃; Yield: 81%. ^1^H NMR (400 MHz, DMSO) *δ* 10.22 (s, 1H, -NH-), 8.01 – 7.92 (m, 4H, Ph-H), 7.86 (ddd, *J* = 7.9, 1.7, 1.0 Hz, 1H, Ph-H), 7.71 (t, *J* = 8.0 Hz, 1H, Ph-H), 7.39 – 7.33 (m, 1H, Ph-H), 7.31 – 7.26 (m, 2H, Ph-H), 7.16 (t, *J* = 8.2 Hz, 2H, Ph-H), 4.41 (s, 2H, Ph-H). ^13^C NMR (100 MHz, DMSO) *δ* 166.76, 165.68, 165.41, 158.85 (d, *J* = 5.2 Hz), 156.38 (d, *J* = 5.3 Hz), 150.51, 135.78, 135.45, 134.65, 132.00, 129.47, 128.65, 128.35 (t, *J* = 10.0 Hz), 127.72, 127.22, 123.39, 114.04 (t, *J* = 17.1 Hz), 112.08, 111.90, 37.12. HRMS (ESI) calcd for C_21_H_14_O_4_N_3_F_2_S_2_ [M-H]^-^: 378.0177, found 378.0182.


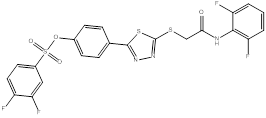
**4-(5-((2-((2,6-difluorophenyl)amino)-2-oxoethyl)thio)-1,3,4-thiadiazol-2-yl)phenyl-3,4-difluorobenzenesulfonate(C_5_)**: Yellow solid, m.p.156-158 ℃; Yield: 87%. ^1^H NMR (500 MHz, DMSO) *δ* 10.20 (s, 1H, -NH-), 8.23 – 8.15 (m, 2H, Ph-H), 8.00 – 7.91 (m, 2H, Ph-H), 7.80 – 7.73 (m, 1H, Ph-H), 7.40 – 7.33 (m, 2H, Ph-H), 7.31 – 7.26 (m, 2H, Ph-H), 7.16 (t, *J* = 8.2 Hz, 1H, Ph-H), 4.42 (s, 2H, -CH_2_-). ^13^C NMR (125 MHz, DMSO) *δ* 166.77, 165.68, 165.40, 158.61 (d, *J* = 5.0 Hz), 156.62 (d, *J* = 5.1 Hz), 154.81 (d, *J* = 12.1 Hz), 152.77 (d, *J* = 12.4 Hz), 150.74 (d, *J* = 13.4 Hz), 150.49, 148.73 (d, *J* = 13.6 Hz), 130.82, 129.46, 128.68, 128.34 (t, *J* = 9.6 Hz), 126.91 (d, *J* = 5.0 Hz), 123.48, 119.44 (d, *J* = 18.8 Hz), 118.68, 118.52, 114.04 (t, *J* = 17.0 Hz), 112.08, 111.90, 37.12. HRMS (ESI) calcd for C_21_H_14_O_4_N_3_F_2_S_2_ [M-H]^-^: 553.9920, found 553.9927.


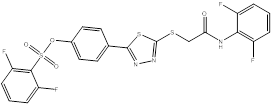
**4-(5-((2-((2,6-difluorophenyl)amino)-2-oxoethyl)thio)-1,3,4-thiadiazol-2-yl)phenyl-2,6-difluorobenzenesulfonate(C_6_)**: Yellow solid, m.p.140-142 ℃; Yield: 85%. ^1^H NMR (500 MHz, DMSO) *δ* 10.20 (s, 1H, -NH-), 8.03 – 7.89 (m, 3H, Ph-H), 7.45 (t, *J* = 8.8 Hz, Ph-H), 7.39 – 7.31 (m, 3H, Ph-H), 7.16 (t, *J* = 8.2 Hz, 2H, Ph-H), 4.42 (s, 2H, -CH_2_-). ^13^C NMR (125 MHz, DMSO) *δ* 166.66, 165.68, 165.48, 160.10 (d, *J* = 2.3 Hz), 158.60 (d, *J* = 5.1 Hz), 158.03 (d, *J* = 2.5 Hz), 156.62 (d, *J* = 5.1 Hz), 150.26, 138.99 (t, *J* = 11.3 Hz), 129.63, 128.93, 128.34 (t, *J* = 9.7 Hz), 122.93, 114.31, 114.10, 112.08, 111.90, 37.12. HRMS (ESI) calcd for C_21_H_14_O_4_N_3_F_2_S_2_ [M-H]^-^: 553.9920, found 553.9928.


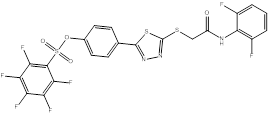
**4-(5-((2-((2,6-difluorophenyl)amino)-2-oxoethyl)thio)-1,3,4-thiadiazol-2-yl)phenyl-pentafluorobenzenesulfonate(C_7_)**: Yellow solid, m.p.140-142 ℃; Yield: 87%. ^1^H NMR (500 MHz, DMSO) *δ* 10.22 (s, 1H, -NH-), 8.06 – 7.95 (m, 2H, Ph-H), 7.52 – 7.44 (m, 2H, Ph-H), 7.36 (ddd, *J* = 14.7, 8.3, 6.3 Hz, 1H, Ph-H), 7.17 (t, *J* = 8.2 Hz, 2H, Ph-H), 4.42 (s, 2H. -CH_2_-). ^13^C NMR (125 MHz, DMSO) *δ* 166.62, 165.64 (d, *J* = 7.1 Hz), 158.61 (d, *J* = 5.0 Hz), 156.63 (d, *J* = 5.1 Hz), 150.06, 129.71, 129.31, 128.34 (t, *J* = 9.9 Hz), 123.31, 114.04 (t, *J* = 16.8 Hz), 112.08, 111.90, 37.13. HRMS (ESI) calcd for C_21_H_14_O_4_N_3_F_2_S_2_ [M-H]^-^: 607.96380, found 607.9646.


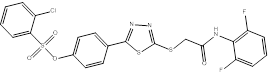
**4-(5-((2-((2,6-difluorophenyl)amino)-2-oxoethyl)thio)-1,3,4-thiadiazol-2-yl)phenyl-2-chlorobenzenesulfonate(C_8_)**: Reddish brown solid, m.p.145-147 ℃; Yield: 83%. ^1^H NMR (500 MHz, DMSO) *δ* 10.21 (s, 1H, -NH-), 8.00 – 7.92 (m, 3H, Ph-H), 7.90 (dd, *J* = 8.1, 1.2 Hz, 1H, Ph-H), 7.83 (td, *J* = 7.7, 1.6 Hz, 1H, Ph-H), 7.58 (td, *J* = 7.7, 1.3 Hz, 1H, Ph-H), 7.36 (ddd, *J* = 14.8, 8.4, 6.3 Hz, 1H, Ph-H), 7.32 – 7.27 (m, 2H, Ph-H), 7.16 (t, *J* = 8.2 Hz, 2H, Ph-H), 4.41 (s, 2H, -CH_2_-). ^13^C NMR (125 MHz, DMSO) *δ* 166.68, 165.67, 165.40, 158.59 (d, *J* = 5.0 Hz), 156.61 (d, *J* = 4.9 Hz), 150.44, 136.91, 132.72, 132.45, 131.91 (d, *J* = 8.0 Hz), 129.56, 128.66, 128.42, 128.34, 123.01, 114.03 (t, *J* = 16.8 Hz), 112.08, 111.90, 37.11. HRMS (ESI) calcd for C_21_H_14_O_4_N_3_F_2_S_2_ [M-H]^-^: 551.9719, found 551.9728.


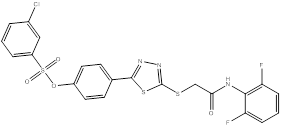
**4-(5-((2-((2,6-difluorophenyl)amino)-2-oxoethyl)thio)-1,3,4-thiadiazol-2-yl)phenyl-3-chlorobenzenesulfonate(C_9_)**: Yellow solid, m.p.131-133 ℃; Yield: 77%. ^1^H NMR (500 MHz, DMSO) *δ* 10.21 (s, 1H, -NH-), 7.99 (t, *J* = 1.9 Hz, 1H, Ph-H), 7.98 – 7.91 (m, 1H, Ph-H), 7.86 (ddd, *J* = 7.9, 1.8, 1.0 Hz, 1H, Ph-H), 7.71 (t, *J* = 8.0 Hz, 1H, Ph-H), 7.36 (ddd, *J* = 14.7, 8.4, 6.3 Hz, 1H, Ph-H), 7.31 – 7.26 (m, 2H, Ph-H), 7.16 (t, *J* = 8.2 Hz, 2H, Ph-H), 4.42 (s, 2H, -CH_2_-). ^13^C NMR (125 MHz, DMSO) *δ* 166.75, 165.67, 165.39, 158.60 (d, *J* = 4.9 Hz), 156.62 (d, *J* = 5.1 Hz), 150.50, 135.78, 135.43, 134.64, 131.98, 129.45, 128.65, 128.34 (t, *J* = 9.7 Hz), 127.72, 127.21, 123.38, 114.04 (t, *J* = 16.8 Hz), 112.08, 111.90, 37.12. HRMS (ESI) calcd for C_21_H_14_O_4_N_3_F_2_S_2_ [M-H]^-^: 551.9719, found 551.9727.


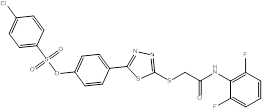
**4-(5-((2-((2,6-difluorophenyl)amino)-2-oxoethyl)thio)-1,3,4-thiadiazol-2-yl)phenyl-4-chlorobenzenesulfonate(C_10_)**: Yellow solid, m.p.159-161 ℃; Yield: 85%. ^1^H NMR (500 MHz, DMSO) *δ* 10.22 (s, 1H, -NH-), 7.93 (dd, *J* = 19.5, 7.9 Hz, 4H, Ph-H), 7.76 (d, *J* = 7.8 Hz, 2H, Ph-H), 7.42 – 7.32 (m, 1H, Ph-H), 7.29 – 7.13 (m, 4H, Ph-H), 4.41 (s, 2H, -CH_2_-). ^13^C NMR (125 MHz, ) *δ* 166.77, 165.68, 165.38, 158.60 (d, *J* = 4.5 Hz), 156.62 (d, *J* = 4.5 Hz), 150.56, 140.44, 132.7, 130.28 (d, *J* = 11.5 Hz), 129.45, 128.61, 128.34 (t, *J* = 9.3 Hz), 123.43, 114.04 (t, *J* = 16.8 Hz), 112.09, 111.90, 37.12. HRMS (ESI) calcd for C_21_H_14_O_4_N_3_F_2_S_2_ [M-H]^-^: 551.9719, found 551.9728.


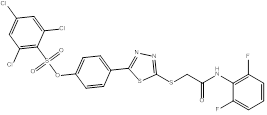
**4-(5-((2-((2,6-difluorophenyl)amino)-2-oxoethyl)thio)-1,3,4-thiadiazol-2-yl)phenyl-2,4,6-trichlorobenzenesulfonate(C_11_)**: Yellow solid, m.p.123-125 ℃; Yield: 71%. ^1^H NMR (500 MHz, DMSO) *δ* 10.21 (s, 1H, -NH-), 8.06 (s, 2H, Ph-H), 8.01 – 7.94 (m, 2H, Ph-H), 7.38 – 7.33 (m, 3H, Ph-H), 7.16 (t, *J* = 8.2 Hz, 2H, Ph-H), 4.42 (s, 2H, -CH_2_-). ^13^C NMR (125 MHz, DMSO) *δ* 166.66, 165.67, 165.47, 158.60 (d, *J* = 5.0 Hz), 156.62 (d, *J* = 5.2 Hz), 150.20, 140.23, 136.18, 131.99, 129.67, 129.28, 128.88, 128.33 (t, *J* = 9.7 Hz), 122.93, 114.04 (t, *J* = 17.0 Hz), 112.08, 111.90, 37.13. HRMS (ESI) calcd for C_21_H_14_O_4_N_3_F_2_S_2_ [M-H]^-^: 619.8940, found 619.8951.


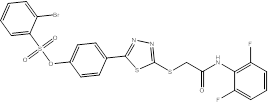
**4-(5-((2-((2,6-difluorophenyl)amino)-2-oxoethyl)thio)-1,3,4-thiadiazol-2-yl)phenyl-2-bromobenzenesulfonate(C_12_)**: Yellow solid, m.p.136-138 ℃; Yield:88%. ^1^H NMR (500 MHz, DMSO) *δ* 10.21 (s, 1H, -NH-), 8.06 (dd, *J* = 8.0, 1.1 Hz, 1H, Ph-H), 8.02 – 7.91 (m, 3H, Ph-H), 7.72 (td, *J* = 7.7, 1.7 Hz, 1H, Ph-H), 7.61 (td, *J* = 7.7, 1.1 Hz, 1H, Ph-H), 7.41 – 7.28 (m, 3H, Ph-H), 7.16 (t, *J* = 8.2 Hz, 2H, Ph-H), 4.41 (s, 2H, -CH_2_-). ^13^C NMR (125 MHz, DMSO) *δ* 166.69, 165.67, 165.39, 158.59 (d, *J* = 4.9 Hz), 156.61 (d, *J* = 5.2 Hz), 150.47, 136.72, 136.23, 133.79, 132.71, 129.55, 128.82, 128.63, 128.34 (t, *J* = 9.8 Hz), 123.04, 120.48, 114.03 (t, *J* = 16.9 Hz), 112.08, 111.90, 37.11. HRMS (ESI) calcd for C_21_H_14_O_4_N_3_F_2_S_2_ [M-H]^-^: 595.9214, found 595.9224.


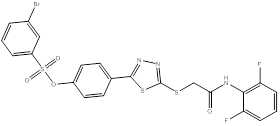
4**-(5-((2-((2,6-difluorophenyl)amino)-2-oxoethyl)thio)-1,3,4-thiadiazol-2-yl)phenyl-3-bromobenzenesulfonate(C_13_)**: Yellow solid, m.p.131-133 ℃; Yield: 76%. ^1^H NMR (500 MHz, DMSO) *δ* 10.21 (s, 1H, -NH-), 8.09 – 8.05 (m, 2H, Ph-H), 8.00 – 7.89 (m, 3H, Ph-H), 7.64 (t, *J* = 7.9 Hz, 1H, Ph-H), 7.36 (ddd, *J* = 14.8, 8.4, 6.3 Hz, 1H, Ph-H), 7.31 – 7.26 (m, 2H, Ph-H), 7.16 (t, *J* = 8.2 Hz, 2H, Ph-H), 4.42 (s, 2H, -CH_2_-). ^13^C NMR (125 MHz, DMSO) *δ* 166.75, 165.68, 165.40, 158.60 (d, *J* = 5.0 Hz), 156.62 (d, *J* = 5.2 Hz), 150.50, 138.31, 135.88, 132.14, 130.39, 129.45, 128.65, 128.34 (t, *J* = 9.4 Hz), 127.52 123.39, 122.80, 114.04 (t, *J* = 16.8 Hz), 112.09, 111.90, 37.12. HRMS (ESI) calcd for C_21_H_14_O_4_N_3_F_2_S_2_ [M-H]^-^: 595.9214, found 595.9226.


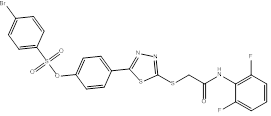
**4-(5-((2-((2,6-difluorophenyl)amino)-2-oxoethyl)thio)-1,3,4-thiadiazol-2-yl)phenyl-4-bromobenzenesulfonate(C_14_)**: Yellow solid, m.p.138-140 ℃; Yield: 81%. ^1^H NMR (500 MHz, DMSO) *δ* 10.21 (s, 1H, -NH-), 7.97 – 7.89 (m, 4H, Ph-H), 7.84 – 7.80 (m, 2H, Ph-H), 7.36 (ddd, *J* = 12.8, 8.4, 6.3 Hz, 1H, Ph-H), 7.28 – 7.23 (m, 2H, Ph-H), 7.17 (t, *J* = 8.2 Hz, 2H, Ph-H), 4.42 (s, 2H, -CH_2_-). ^13^C NMR (125 MHz, DMSO) *δ* 166.76, 165.67, 165.37, 158.60 (d, *J* = 4.8 Hz), 156.61 (d, *J* = 5.0 Hz), 150.56, 133.19 (d, *J* = 5.9 Hz), 130.27, 129.67, 129.45, 128.60, 128.34 (t, *J* = 9.7 Hz), 123.42, 114.04 (t, *J* = 17.0 Hz), 112.09, 111.90, 37.12. HRMS (ESI) calcd for C_21_H_14_O_4_N_3_F_2_S_2_ [M-H]^-^: 595.9214, found 595.9224.


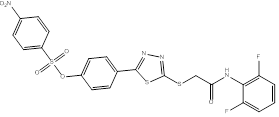
**4-(5-((2-((2,6-difluorophenyl)amino)-2-oxoethyl)thio)-1,3,4-thiadiazol-2-yl)phenyl-4-nitrobenzenesulfonate(C_15_)**: Yellow solid, m.p.196-198 ℃; Yield: 82%. ^1^H NMR (500 MHz, DMSO) *δ* 10.21 (s, 1H, -NH-), 8.48 – 8.44 (m, 2H, Ph-H), 8.21 – 8.17 (m, 2H, Ph-H), 7.99 – 7.90 (m, 2H, Ph-H), 7.36 (ddd, *J* = 14.8, 8.4, 6.3 Hz, 2H, Ph-H), 7.30 – 7.25 (m, 2H, Ph-H), 7.16 (t, *J* = 8.2 Hz, 2H, Ph-H), 4.41 (s, 2H, -CH_2_-). ^13^C NMR (125 MHz, DMSO) *δ* 166.69, 165.67, 165.46, 158.60 (d, *J* = 5.0 Hz), 156.61 (d, *J* = 5.1 Hz), 151.23, 150.38, 139.17, 130.20, 129.53, 128.80, 128.34 (t, *J* = 9.6 Hz), 125.21, 123.43, 114.03 (t, *J* = 16.8 Hz), 112.09, 111.90, 37.11. HRMS (ESI) calcd for C_21_H_14_O_4_N_3_F_2_S_2_ [M-H]^-^: 562.9960, found 562.9970.


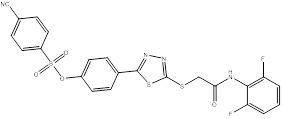
**4-(5-((2-((2,6-difluorophenyl)amino)-2-oxoethyl)thio)-1,3,4-thiadiazol-2-yl)phenyl-4-cyanobenzenesulfonate(C_16_)**: Yellow solid, m.p.162-164 ℃; Yield: 89%. ^1^H NMR (500 MHz, DMSO) *δ* 10.22 (s, 1H, -NH-), 8.20 – 8.16 (m, 2H, Ph-H ), 8.12 – 8.08 (m, 2H, Ph-H), 7.99 – 7.91 (m, 1H, Ph-H), 7.36 (ddd, *J* = 14.8, 8.4, 6.3 Hz, 1H, Ph-H), 7.28 – 7.24 (m, 2H, Ph-H), 7.16 (t, *J* = 8.2 Hz, 2H, Ph-H), 4.42 (s, 2H, -CH_2_-). ^13^C NMR (125 MHz, DMSO) *δ* 166.71, 165.68, 165.44, 158.60 (d, *J* = 5.0 Hz), 156.62 (d, *J* = 4.9 Hz), 150.40, 137.94, 134.10, 129.51, 129.17, 128.75), 128.34 (t, *J* = 9.7 Hz), 123.41, 117.61, 117.34, 114.04 (t, *J* = 16.9 Hz), 112.09, 111.90, 37.12. HRMS (ESI) calcd for C_21_H_14_O_4_N_3_F_2_S_2_ [M-H]^-^: 543.0062, found 543.0070.


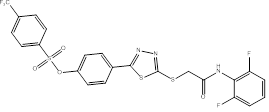
**4-(5-((2-((2,6-difluorophenyl)amino)-2-oxoethyl)thio)-1,3,4-thiadiazol-2-yl)phenyl-4-(trifluoromethyl)benzenesulfonate(C_17_)**: Yellow solid, m.p.176-178 ℃; Yield: 83%. ^1^H NMR (500 MHz, DMSO) *δ* 10.22 (s, 1H, -NH-), 8.14 (d, *J* = 8.3 Hz, 2H, Ph-H), 8.08 (d, *J* = 8.4 Hz, 2H, Ph-H), 8.00 – 7.92 (m, 2H, Ph-H), 7.36 (ddd, *J* = 14.8, 8.5, 6.3 Hz, 1H, Ph-H), 7.30 – 7.26 (m, 2H, Ph-H), 7.16 (t, *J* = 8.2 Hz, 2H, Ph-H), 4.42 (s, 2H, -CH_2_-). ^13^C NMR (125 MHz, DMSO) *δ* 166.72, 165.68, 165.43, 158.60 (d, *J* = 5.2 Hz), 156.62 (d, *J* = 5.2 Hz), 150.47, 137.97, 134.67, 134.41, 129.51, 128.73 128.34 (t, *J* = 9.7 Hz), 127.24 (d, *J* = 3.5 Hz), 124.27, 123.40, 122.10, 114.04 (t, *J* = 17.0 Hz), 112.09, 111.90, 37.12. HRMS (ESI) calcd for C_21_H_14_O_4_N_3_F_2_S_2_ [M-H]^-^: 585.9983, found 585.9992.


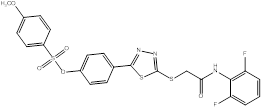
**4-(5-((2-((2,6-difluorophenyl)amino)-2-oxoethyl)thio)-1,3,4-thiadiazol-2-yl)phenyl-4-methoxybenzenesulfonate(C_18_)**: Yellow solid, m.p.144-146 ℃; Yield: 86%. ^1^H NMR (500 MHz, DMSO) *δ* 10.21 (s, 1H, -NH-), 7.97 – 7.90 (m, 2H, Ph-H), 7.83 – 7.79 (m, 2H, Ph-H), 7.36 (ddd, *J* = 14.8, 8.4, 6.3 Hz, 1H, Ph-H), 7.22 – 7.14 (m, 6H, Ph-H), 4.41 (s, 2H, -CH_2_-), 3.86 (s, 3H, -CH_3_). ^13^C NMR (125 MHz, DMSO) *δ* 166.86, 165.69, 165.28, 164.25, 158.60 (d, *J* = 5.0 Hz), 156.62 (d, *J* = 5.1 Hz), 150.8, 130.81, 129.32, 128.32 (d, *J* = 4.8 Hz), 125.13, 123.41, 115.15, 114.04 (t, *J* = 16.8 Hz), 112.09, 111.91, 56.03, 37.12. HRMS (ESI) calcd for C_21_H_14_O_4_N_3_F_2_S_2_ [M-H]^-^: 528.0215, found 548.0222.


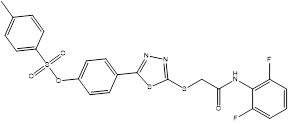
**4-(5-((2-((2,6-difluorophenyl)amino)-2-oxoethyl)thio)-1,3,4-thiadiazol-2-yl)phenyl-4-methylbenzenesulfonate(C_19_)**: Yellow solid, m.p.128-130 ℃; Yield: 86%. ^1^H NMR (500 MHz, DMSO) *δ* 10.22 (s, 1H, -NH-), 7.98 – 7.89 (m, 2H, Ph-H), 7.79 – 7.76 (m, 2H, Ph-H), 7.48 (d, *J* = 8.0 Hz, 2H, Ph-H), 7.36 (ddd, *J* = 14.8, 8.4, 6.3 Hz, 1H, Ph-H), 7.24 – 7.13 (m, 4H, Ph-H), 4.41 (s, 2H, -CH_2_-), 2.41 (s, 3H, -CH_3_). ^13^C NMR (125 MHz, DMSO) *δ* 166.82, 165.68, 165.30, 158.60 (d, *J* = 5.0 Hz), 156.62 (d, *J* = 5.1 Hz), 150.78, 146.19, 131.12, 130.43, 129.34, 128.37, 123.34, 114.04 (t, *J* = 16.8 Hz), 112.09, 111.90, 37.12, 21.26. HRMS (ESI) calcd for C_21_H_14_O_4_N_3_F_2_S_2_ [M-H]^-^: 532.0266, found 532.0272.


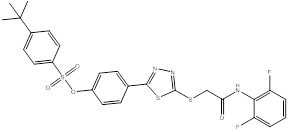
**4-(5-((2-((2,6-difluorophenyl)amino)-2-oxoethyl)thio)-1,3,4-thiadiazol-2-yl)phenyl-4-(tert-butyl)benzenesulfonate(C_20_)**: Reddish brown solid, m.p.58-60 ℃: Yield: 88%. ^1^H NMR (500 MHz, DMSO) *δ* 10.21 (s, 1H, -NH-), 7.99 – 7.91 (m, 2H, Ph-H), 7.86 – 7.82 (m, 2H, Ph-H), 7.72 – 7.68 (m, 2H, Ph-H), 7.36 (ddd, *J* = 14.7, 8.4, 6.3 Hz, 1H, Ph-H), 7.27 – 7.21 (m, 2H, Ph-H), 7.16 (t, *J* = 8.2 Hz, 2H, Ph-H), 4.41 (s, 2H, -CH_2_-), 1.30 (s, 9H, -C(CH_3_)_3_). ^13^C NMR (125 MHz, DMSO) *δ* 166.83, 165.68, 165.32, 158.59, 156.62 (d, *J* = 5.2 Hz), 150.77, 131.36, 129.36, 128.29 (d, *J* = 15.2 Hz), 126.88, 123.27, 114.04 (t, *J* = 16.9 Hz), 112.09, 111.90, 37.12, 35.25, 30.68. HRMS (ESI) calcd for C_21_H_14_O_4_N_3_F_2_S_2_ [M-H]^-^: 574.0735, found 574.0742.

^1^H NMR of compound A-1

^13^C NMR of compound A-1

 HRMS of compound A-1

^1^H NMR of compound A_1_

^13^C NMR of compound A_1_

HRMS of compound A_1_

^1^H NMR of compound A_2_

^13^C NMR of compound A_2_

 HRMS of compound A_2_

^1^H NMR of compound A_3_

^13^C NMR of compound A_3_

 HRMS of compound A_3_

^1^H NMR of compound A_4_

^13^C NMR of compound A_4_

 HRMS of compound A_4_

^1^H NMR of compound A_5_

^13^C NMR of compound A_5_

 HRMS of compound A_5_

^1^H NMR of compound A_6_

^13^C NMR of compound A_6_

HRMS of compound A_6_

^1^H NMR of compound A_7_

^13^C NMR of compound A_7_

HRMS of compound A_7_

^1^H NMR of compound A_8_

^13^C NMR of compound A_8_

HRMS of compound A_8_

^1^H NMR of compound A_9_

^13^C NMR of compound A_9_

HRMS of compound A_9_

^1^H NMR of compound A_10_

^13^C NMR of compound A_10_

HRMS of compound A_10_

^1^H NMR of compound A_11_

^13^C NMR of compound A_11_

HRMS of compound A_11_

^1^H NMR of compound A_12_

^13^C NMR of compound A_12_

HRMS of compound A_12_

^1^H NMR of compound A_13_

^13^C NMR of compound A_13_

HRMS of compound A_13_

^1^H NMR of compound A_14_

^13^C NMR of compound A_14_

HRMS of compound A_14_

^1^H NMR of compound A_15_

HRMS of compound A_15_

^1^H NMR of compound A_16_

^13^C NMR of compound A_16_

HRMS of compound A_16_

^1^H NMR of compound A_17_

^13^C NMR of compound A_17_

HRMS of compound A_17_

^1^H NMR of compound A_18_

^13^C NMR of compound A_18_

HRMS of compound A_18_

^1^H NMR of compound B-3

^13^C NMR of compound B-3

 HRMS of compound B-3

^1^H NMR of compound B_1_

^13^C NMR of compound B_1_

HRMS of compound B_1_

^1^H NMR of compound B_2_

^13^C NMR of compound B_2_

HRMS of compound B_2_

^1^H NMR of compound B_3_

^13^C NMR of compound B_3_

HRMS of compound B_3_

^1^H NMR of compound B_4_

^13^C NMR of compound B_4_

HRMS of compound B_4_

^1^H NMR of compound B_5_

^13^C NMR of compound B_5_

HRMS of compound B_5_

^1^H NMR of compound B_6_

^13^C NMR of compound B_6_

HRMS of compound B_6_

^1^H NMR of compound B_7_

^13^C NMR of compound B_7_

HRMS of compound B_7_

^1^H NMR of compound B_8_

^13^C NMR of compound B_8_

HRMS of compound B_8_

^1^H NMR of compound B_9_

^13^C NMR of compound B_9_

HRMS of compound B_9_

^1^H NMR of compound B_10_

^13^C NMR of compound B_10_

HRMS of compound B_10_

^1^H NMR of compound B_11_

^13^C NMR of compound B_11_

HRMS of compound B_11_

^1^H NMR of compound B_12_

^13^C NMR of compound B_12_

HRMS of compound B_12_

^1^H NMR of compound B_13_

^13^C NMR of compound B_13_

HRMS of compound B_13_

^1^H NMR of compound B_14_

^13^C NMR of compound B_14_

HRMS of compound B_14_

^1^H NMR of compound B_15_

^13^C NMR of compound B_15_

HRMS of compound B_15_

^1^H NMR of compound B_16_

^13^C NMR of compound B_16_

HRMS of compound B_16_

^1^H NMR of compound B_17_

^13^C NMR of compound B_17_

HRMS of compound B_17_

^1^H NMR of compound B_18_

^13^C NMR of compound B_18_

HRMS of compound B_18_

^1^H NMR of compound B_19_

^13^C NMR of compound B_19_

HRMS of compound B_19_

^1^H NMR of compound B_20_

^13^C NMR of compound B_20_

HRMS of compound B_20_

^1^H NMR of compound C-4

^13^C NMR of compound C-4

HRMS of compound C-4

^1^H NMR of compound C_1_

^13^C NMR of compound C_1_

HRMS of compound C_1_

^1^H NMR of compound C_2_

^13^C NMR of compound C_2_

HRMS of compound C_2_

^1^H NMR of compound C_3_

^13^C NMR of compound C_3_

HRMS of compound C_3_

^1^H NMR of compound C_4_

^13^C NMR of compound C_4_

HRMS of compound C_4_

^1^H NMR of compound C_5_

^13^C NMR of compound C_5_

HRMS of compound C_5_

^1^H NMR of compound C_6_

^13^C NMR of compound C_6_

HRMS of compound C_6_

^1^H NMR of compound C_7_

^13^C NMR of compound C_7_

HRMS of compound C_7_

^1^H NMR of compound C_8_

^13^C NMR of compound C_8_

HRMS of compound C_8_

^1^H NMR of compound C_9_

^13^C NMR of compound C_9_

HRMS of compound C_9_

^1^H NMR of compound C_10_

^13^C NMR of compound C_10_

HRMS of compound C_10_

^1^H NMR of compound C_11_

^13^C NMR of compound C_11_

HRMS of compound C_11_

^1^H NMR of compound C_12_

^13^C NMR of compound C_12_

HRMS of compound C_12_

^1^H NMR of compound C_13_

^13^C NMR of compound C_13_

HRMS of compound C_13_

^1^H NMR of compound C_14_

^13^C NMR of compound C_14_

HRMS of compound C_14_

^1^H NMR of compound C_15_

^13^C NMR of compound C_15_

HRMS of compound C_15_

^1^H NMR of compound C_16_

^13^C NMR of compound C_16_

HRMS of compound C_16_

^1^H NMR of compound C_17_

^13^C NMR of compound C_17_

HRMS of compound C_17_

^1^H NMR of compound C_18_

^13^C NMR of compound C_18_

HRMS of compound C_18_

^1^H NMR of compound C_19_

^13^C NMR of compound C_19_

HRMS of compound C_19_

^1^H NMR of compound C_20_

^13^C NMR of compound C_20_

HRMS of compound C_20_
